# Polymeric microneedles enable simultaneous delivery of cancer immunomodulatory drugs and detection of skin biomarkers

**DOI:** 10.7150/thno.73966

**Published:** 2023-01-01

**Authors:** Pere Dosta, Núria Puigmal, Alexander M. Cryer, Alma L. Rodríguez, Ella Scott, Ralph Weissleder, Miles A. Miller, Natalie Artzi

**Affiliations:** 1 Institute for Medical Engineering and Science (IMES), Massachusetts Institute of Technology, Cambridge, MA 02139.; 2 Department of Medicine, Division of Engineering in Medicine, Brigham and Women's Hospital, Harvard Medical School, Boston, MA 02115.; 3 Center for Systems Biology, Massachusetts General Hospital Research Institute, Boston, MA 02114.; 4 Department of Radiology, Massachusetts General Hospital, Harvard Medical School, Boston, MA 02114.; 5 Department of Systems Biology, Harvard Medical School.; 6 Wyss Institute for Biologically Inspired Engineering, Harvard University, Boston, MA, 02115.

**Keywords:** microneedles, immunotherapy, cancer, poly(beta-amino ester)s, theranostics

## Abstract

**Background:** Immune-modulating therapies impart positive outcomes in a subpopulation of cancer patients. Improved delivery strategies and non-invasive monitoring of anti-tumor effects can help enhance those outcomes and understand the mechanisms associated with the generation of anti-tumor immune responses following immunotherapy.

**Methods:** We report on the design of a microneedle (MN) platform capable of simultaneous delivery of immune activators and collection of interstitial skin fluid (ISF) to monitor therapeutic responses. While either approach has shown promise, the integration of the therapy and diagnostic arms into one MN platform has hardly been explored before. MNs were synthesized out of crosslinked hyaluronic acid (HA) and loaded with a model immunomodulatory nanoparticle-containing drug, CpG oligodinucleotides (TLR9 agonist), for cancer therapy in melanoma and colon cancer models. The therapeutic response was monitored by longitudinal analysis of entrapped immune cells in the MNs following patch retrieval and digestion.

**Results:** Transdermal delivery of CpG-containing NPs with MNs induced anti-tumor immune responses in multiple syngeneic mouse cancer models. CpG-loaded MNs stimulated innate immune cells and reduced tumor growth. Intravital microscopy showed deposition and spatiotemporal co-localization of CpG-NPs within the tumor microenvironment when delivered with MNs. Analysis of MN-sampled ISF revealed similar immune signatures to those seen in the bulk tumor homogenate, such as increased populations of macrophages and effector T cells following treatment.

**Conclusions:** Our hydrogel-based MNs enable effective transdermal drug delivery into immune cells in the tumor microenvironment, and upon retrieval, enable studying the immune response to the therapy over time. This platform has the theranostic potential to deliver a range of combination therapies while detecting biomarkers.

## Introduction

Microneedles (MNs) have emerged as promising platforms in the drug delivery field, enabling self-administration, needle-free penetration, and minimally invasive access to a complex immune milieu unique to the skin 1,2. MNs have been used to deliver anti-tumoral molecules (such as chemotherapeutics, cytokines, and nucleic acids) either in the form of free drugs or as nano-formulations 3,4. Applying these paradigms for transdermal delivery of immune therapy is particularly intriguing 5,6 owing to facile access to immune cells and the potential to avoid detrimental systemic toxicities 6-8. MNs have also been recently investigated for minimally invasive extraction of interstitial skin fluid (ISF) to permit early detection and clinical monitoring of a wide range of pathologies, including cancer 9-13. ISF comprises 75% of all extracellular fluid and contains biomarkers that can reflect physiological function and correlate with disease state 14-17. Surveillance of immune infiltrates and soluble biomarkers in the tumor microenvironment (TME) has been proposed to assess responsiveness to immunotherapy 11,18, yet there is little data on whether this is of clinical value. In breast cancer models, such correlation has been reported 19-21, prompting us to hypothesize that the use of ISF for diagnostic purposes could have greater translational potential when targeting superficial tumors such as melanoma, which allow for easy access with minimally invasive platforms such as MNs.

Despite their promise for therapeutic and diagnostic purposes, MNs encompassing both abilities within the same platform —as a theranostic tool— have rarely been explored. Combining both compartments requires designs that allow for high drug loading and maximal sampling volume of biomarkers. MNs have been recently designed to detect soluble biomarkers such as nucleic acids 11, hormones 22, metabolites 13,23,24, or viral capsids 12. Some of these designs often require harsh downstream processing to recover the biomarkers (including high-speed centrifugation 24 or heat treatments 23), which makes them incompatible with cell sampling. Instead, to efficiently isolate cells and cellular biomarkers that have accumulated in MNs, dissolvable MNs are needed to release their contents for further analysis while maintaining cell viability and phenotype 25,26. Here, we present the design and application of an adjuvant-loaded hydrogel-based MN platform for cancer theranostics. This device delivers an immunomodulator to augment anti-cancer immunity while subsequently sampling cell fractions from the ISF for surveillance of the TME in response to the therapy.

Unmethylated cytosine-phosphate-guanine (CpG) oligonucleotides (ODNs) are well-described innate immune effectors capable of instigating potent anti-tumor responses by selectively engaging toll-like receptors (TLRs) expressed by innate immune cells 27,28. However, *in vivo* delivery of CpG continues to be challenging due to high hydrophilicity and rapid degradation 29. To overcome these limitations, our theranostic MNs were loaded with nanoparticles (NPs) derived from poly (β-amino ester)s (PBAEs) 30-34 that encapsulate the CpG-ODNs (herein referred to as CpG-NPs) to protect them from degradation and enhance cellular internalization 29,35. Here, we demonstrate that transdermal delivery of CpG-NPs via MNs alters the functional state of the tumor immune infiltrate, reduces tumor growth, and increases overall survival in syngeneic melanoma and colorectal murine cancer models. Intravital microscopy (IVM) confirmed the ability of the MNs to localize CpG-NPs inside the tumor. In addition, the MNs permitted subsequent longitudinal ISF sampling of the dermal fraction of the tumor. Analysis of the cellular biomarkers in the ISF confirmed a correlation with the immune changes observed in the TME in response to the immunotherapy, therefore establishing a basis for using MNs as a theranostic platform for simultaneously perturbing the TME and monitoring such disturbances in response to the therapy.

## Results and Discussion

### Engineered hydrogel-based MNs for cancer theranostics

**Figure 1:**
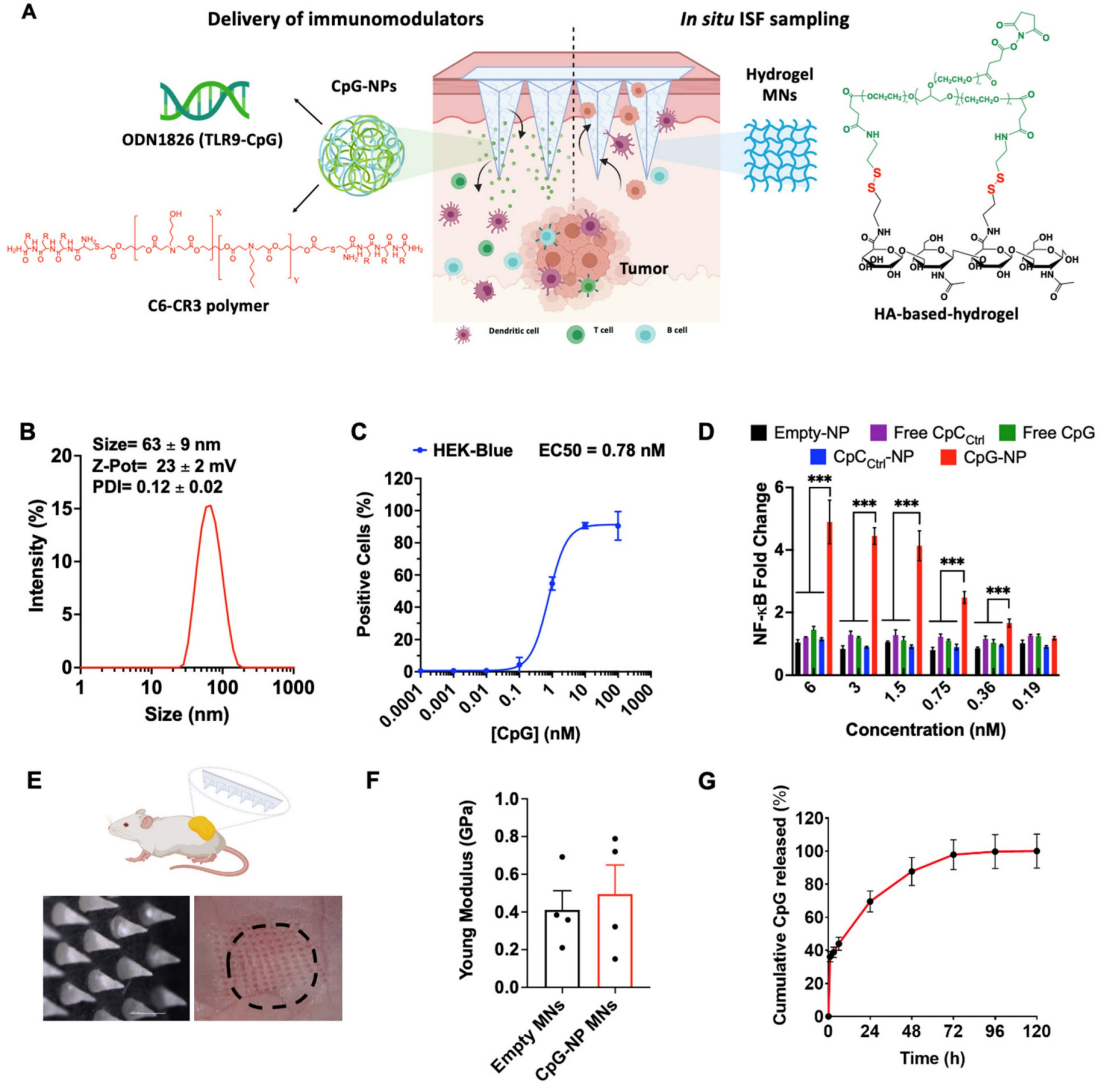
** Engineering a theranostic microneedle platform for management of skin cancer. A,** Representative scheme of a hyaluronic acid (HA)-based microneedle platform for the delivery of immunomodulatory drugs (CpG-ODNs) complexed with poly (beta-amino ester)s (PBAEs) and simultaneous sampling of interstitial fluid (ISF) for recovery of immune cells *ex vivo*. **B,** Biophysical characterization of CpG-containing nanoparticles by dynamic light scattering. **C,** Quantification by flow cytometry of the cellular internalization of CpG-NPs by TLR9-expressing HEK 293 cells (n = 3 biologically independent samples). **D,** Dose-response of NF-kB produced by free CpG, free CpC_Ctrl_, CpG-NPs, CpC_Ctrl_-NPs and empty-NPs in HEK293 TLR9 reporter cell line *in vitro* (n = 4 biologically independent samples). **E,** Microscopy image of the HA-based MNs (scale bar = 500 µm) and representative image of mouse skin after *in vivo* administration of hydrogel MNs into the tumor. The dotted area indicates the tumor site. **F,** Characterization of the mechanical properties of HA-based MNs. A compression test was performed to compare the mechanical strength of empty MNs versus CpG-NP-loaded MNs. Data are mean ± s.e.m. (n = 4). **G,** *In vitro* CpG-NP release profile from the MNs assessed by tracking the fluorescence intensity of labeled NPs over time (pH = 7.4, 37 °C). Data are mean ± s.e.m. (n = 4).

In this study, we aimed to leverage the theragnostic potential of our Hyaluronic Acid-based MN platform for anti-cancer purposes by (1) delivering nano-encapsulated drugs in the vicinity of tumors to suppress tumor growth while simultaneously (2) sampling ISF from the TME to monitor the response to the therapy (**Figure 1A**).

To construct the MNs, the HA polymeric backbone was modified with cysteamine dihydrochloride to include primary amine groups for hydrogel formation and disulfide bonds for reduction-mediated hydrogel digestion using tris (2-carboxyethyl) phosphine (TCEP) (**Figure S1**). To form the MNs, the modified HA polymer containing primary amines was crosslinked with 8-Arm-NHS-polyethylene glycol (PEG) containing a succinimidyl functional group, allowing for spontaneous hydrogel formation (**Figure S2**). Once the MN hydrogel backbone was formed, the nano-encapsulated drugs were incorporated by centrifugation, and the MN patch was finalized by adding a poly lactic-co-glycolic acid (PLGA) back layer. The resulting MNs could be digested on-demand in less than 5 min when incubated with a 10 mM TCEP solution (Figure S3A-B), as determined morphologically by microscopy (Figure S3C). To further confirm MN digestibility, fluorescently labeled MNs fabricated with AF647-conjugated HA were incubated with either the TCEP solution or PBS. Next, the supernatants were collected and the levels of fluorescence were analyzed. Here, we observed an 8-fold increase in fluorescence signal owing to the release of labeled HA from the bulk MN structure when incubated with TCEP, whereas no significant release was observed when incubated in PBS (Figure S3D).

TLR9 agonists, including CpG ODN 1826, were used as a model agent to prove the therapeutic merit of the MN platform as a delivery device and to monitor the TME response to the therapy. TLR9 agonists have been shown to inhibit tumor growth in pre-clinical studies with well-described mechanisms of action 36,37. Yet, their nature - being single-stranded and short synthetic DNA molecules - makes them particularly prone to premature cleavage by endonucleases if delivered in a free form. Given the ability of pBAE polymers to encapsulate and deliver nucleic acids such as plasmid DNA 31,32 microRNA 33, mRNA 38 and siRNA 34, we selected a tri-arginine-modified PBAE polymer (C6-CR3) 30 (**Figure S4 and Figure S5**) as a model delivery vehicle to protect the ODNs and enhance their cellular internalization. To establish the C6-CR3-to-CpG ratio required for the complete complexation of the nucleic acids, we used a gel retardation assay (**Figure S6A**). Here, the presence of cationic groups in the C6-CR3 polymer allowed electrostatic binding to the negatively charged phosphate groups of the CpG-ODNs. The gel retardation assay revealed that CpG-ODNs migration was impeded at weight ratios of 50:1 C6-CR3:CpG. Therefore, we used this formulation throughout the rest of the study. Analysis by dynamic light scattering (DLS) confirmed the encapsulation of the CpG into monodisperse particles (PDI = 0.12 ± 0.02) with a hydrodynamic diameter of 63 ± 9 nm. (**Figure 1A**) that remained relatively unchanged for more than a week at room temperature (**Figure S6B**). Analysis of the particles via transmission electron microscopy (TEM) confirmed their sphere-like morphology and size as per DLS measurements (**Figure S7**). CpG-NPs were positively charged (zeta potential = 23 ± 2 mV) attributed to the high density of protonated groups in the polypeptides (**Figure 1B**).

We next investigated the ability of CpG-NPs to be internalized by murine TLR9-reporter HEK 293 cells (**Figure 1C**). Cells were incubated with varying concentrations of fluorescent CpG-NPs (from 0.0001 nM to 1000 nM) for 24 h in a complete growth medium, confirming over 90% of NP internalization at 10 nM and half-maximal effective concentration (EC50) of 0.78 nM. We also studied the ability of CpG-NPs to stimulate the NF-κB response, being the central signaling pathway for the induction of pro-inflammatory responses 39. Following 24 h incubation with HEK293 cells expressing murine TLR9, we observed that the CpG-NPs elicited the highest activation, with up to 4-fold increase compared to free CpG (**Figure 1D**). The NF-κB response was explicitly triggered by the CpG-NPs, as negative control NPs (CpC_Ctrl_-NPs, harboring CpC instead of CpG motifs that cannot bind and activate TLR receptors) formulated with the same polymer —the tri-arginine-modified PBAE polymer (C6-CR3)— and with comparable features in terms of size and surface charge (**Table S1**), yielded negligible activation levels. In addition, we confirmed that the CpG-NPs remained biologically active once released from the MNs (**Figure S8**). Finally, cytotoxicity of CpG-NPs was studied by MTS cell proliferation assay using HEK293 TLR9 reporter cells. CpG-NP cytotoxicity (**Figure S9**) was only observed at ~20-fold higher concentration (>150nM) than that required to stimulate TLR9-dependent NF-kB transcriptional activity (less than 6 nM) (**Figure 1D**).

We next examined the properties of the theranostic MN platform and its behavior when integrating the NPs into the hydrogel matrix, forming the MNs for delivery into the dermal milieu surrounding the tumors. Here, we proposed the use of MNs with a length of 600 µm and a base width of 300 µm which others have demonstrated to allow for transdermal drug delivery in superficial skin cancers 40-42. We confirmed that MNs could efficiently disrupt the stratum corneum in the tumor area in mice (as evidenced by the presence of micro-conduits) (**Figure 1E**). Quantification of the mechanical strength of the MNs by compression testing did not reveal significant differences between empty MN patches and those loaded with NPs (**Figure 1F, Figure S10A**), supporting our previous findings that the inclusion of therapeutics such as chemokines or cytokines in the MN matrix did not have an impact on its physical properties 25. Similar results were observed in terms of swelling capacity, as no significant differences were observed between the empty and the NP-loaded MNs (**Figure S10B**). Lastly, we confirmed the successful release of CpG-NPs from the MNs. We assessed the release kinetics of fluorescently labeled CpG-NPs when incubated under physiologically relevant conditions (pH = 7.4, 37 °C) *in vitro* (**Figure 1G**). We confirmed that 70% of the CpG-NPs were released within the first 24 h of incubation and that a plateau was achieved by 72 h. Release studies *in vivo* showed that half of the NP cargo was delivered from the MNs within 24 h (**Table S2**). MNs retrieved from mice were digested, and the fluorescence associated with the remaining CpG-NPs inside the MN matrix was compared to the baseline levels recorded from MNs prior to implantation. In light of these findings, a total of 2 µg CpG per MN patch was loaded in subsequent *in vivo* studies to reach a therapeutic target of 1 µg CpG per mouse following 24 h-MN administration.

### CpG-NPs enhance the activation of innate immune cells

**Figure 2:**
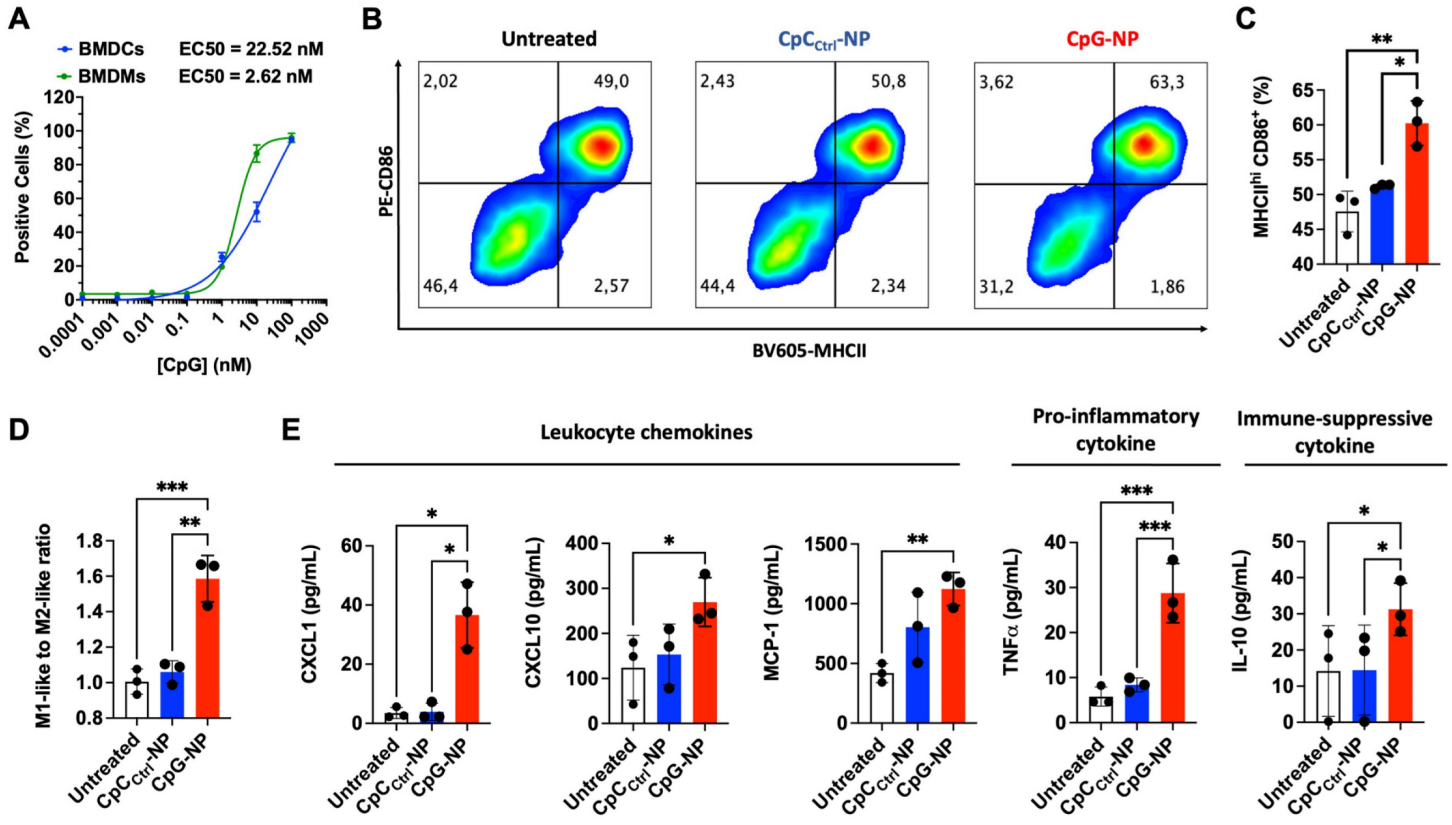
** CpG-NPs activate innate immune cells *in vitro*. A,** Quantification by flow cytometry of the cellular internalization of CpG-NPs by bone marrow-derived dendritic cells (BMDCs) and bone marrow-derived macrophages (BMDMs) *in vitro* (n = 3 biologically independent samples). **B,** Representative flow cytometry density plot of MHCII and CD86 expression in BMDCs treated with CpG-NPs or CpC_Ctrl_-NPs at 10 nM (red denotes higher cell density). **C,** Flow cytometry quantification of MHCII^hi^ CD86^+^ expression in BMDCs 24 h following CpG-NP or CpC_Ctrl_-NP treatment at 10 nM. Data are mean ± s.e.m. (n = 3). **D,** Flow cytometry quantification of CD86^+^ / CD206^+^ (M1-like to M2-like ratio) 24 h following CpG-NP or CpC_Ctrl_-NP treatment at 10 nM, in CD11b^+^ F4/80^+^ cells. Data are mean ± s.e.m. (n = 3). **E,** Protein analysis of BMDM supernatant, 24 h after treatment with CpG-NPs or CpC_Ctrl_-NPs. Data are mean ± s.e.m (n = 3 samples per group). ***P < 0.001, **P < 0.01, *P < 0.05.

We next studied the capability of CpG-NPs to activate murine bone marrow-derived dendritic cells (BMDCs) and bone marrow-derived macrophages (BMDMs). BMDCs and BMDMs were generated following published guidelines 43 that involved culturing isolated murine bone marrow cells in GM-CSF or M-CSF, respectively (see Methods). BMDCs phenotype was confirmed as CD11c^+^ MHCII^+^ and BMDMs phenotype was confirmed as CD11b^+^ F4/80^+^ by flow cytometry. We first studied the ability of BMDMs and BMDCs to efficiently internalize CpG-NPs, and found EC50 values of 2.6 nM and 22.5 nM, respectively (**Figure 2A**). To test the downstream cellular response to CpG-NPs, BMDCs were treated for 24 h with CpG-NPs or CpC_Ctrl_-NPs, and the expression of cell activation markers was determined by flow cytometry. A 10% increase of cells expressing CD86^+^MHCII^hi^ (major histocompatibility complex class II, high expression) was observed in BMDCs treated with CpG-NPs compared to CpC_Ctrl_-NPs. In addition, no significant CD86^+^MHCII^hi^ population increase was observed when comparing the levels of activation in CpC_Ctrl_-NP-treated BMDCs and the untreated ones, corroborating that TLR9-specific activation only occurred in the presence of CpG (**Figure 2B, 2C**). Similar results were observed in BMDMs since an increase in the ratio of pro-inflammatory to anti-inflammatory macrophages was detected when BMDMs were treated with 10 nM of CpG-NPs compared to untreated or CpC_Ctrl_-NP controls (pro-inflammatory M1-like BMDMs were defined as CD86^+^ cells and anti-inflammatory M2-like as CD206^+^ cells) (**Figure 2D**). These results confirmed that CpG-NPs stimulate the maturation and activation of BMDCs and BMDMs. Following TLR engagement, activated BMDMs are known to produce a wide range of cytokines and chemokines to promote anti-tumor immunity 44. To test that, we used an antibody multi-analyte flow assay kit to analyze the supernatant of CpG-NP-treated macrophages. An increase in leukocyte chemoattractants (MCP-1, CXCL1, and CXCL10), as well as pro-inflammatory cytokine (TNF-α), was observed (**Figure 2E**). The immunosuppressive IL-10 cytokine was also upregulated following TLR stimulation, in agreement with the literature 45.

### CpG-NPs suppress tumor growth when delivered via the dual-function MN platform

**Figure 3:**
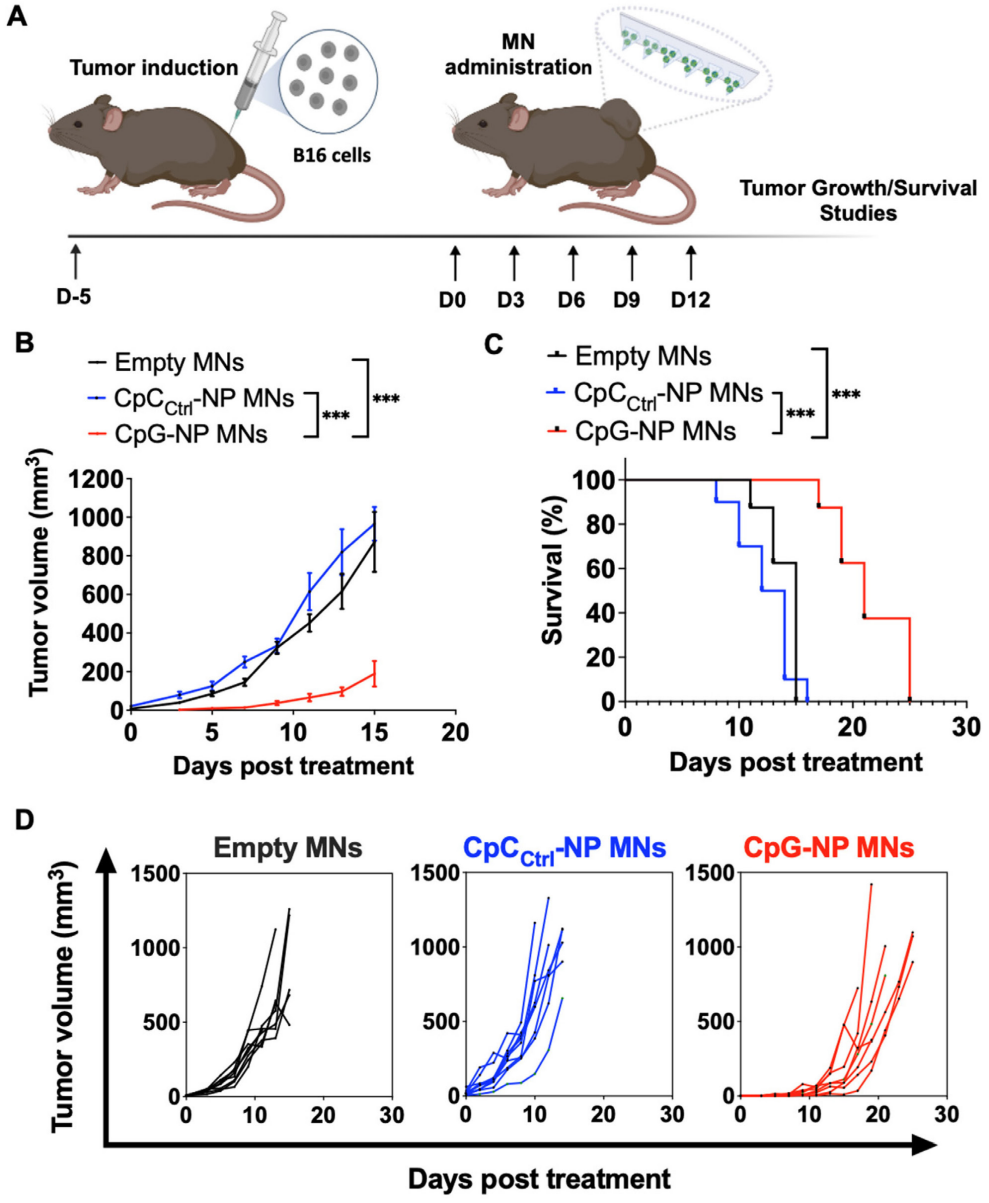
** Transdermal delivery of CpG-NPs using MNs reduces tumor growth and increases survival in a murine model of orthotopic melanoma. A,** Five days post tumor implantation, orthotopic tumors were treated with MNs loaded with CpG-NPs (1 µg CpG-ODN delivered/patch), CpC_Ctrl_-NPs (1 µg CpC-ODN delivered/patch) or empty MNs. MNs were applied on top of the tumor every three days for five cycles (q3dx5). **B**; Tumor growth in B16-F10 melanoma-bearing mice (n = 8-10, data are mean ± s.e.m.). **C,** Kaplan-Meier curves of humane survival. Statistical significance was determined between groups by the Mantel-Cox test. ***P < 0.001, **P < 0.01, *P < 0.05. **D,** Individual tumor growth curves of empty MNs, CpC_Ctrl_-NP MNs, and CpG-NP MNs treatment groups (n = 8-10 biologically independent samples).

Following the characterization of the MN platform, we pursued *in vivo* studies in mice to validate the theranostic ability of the MN platform. We first used an orthotopic murine model of melanoma (B16-F10) as a clinically relevant superficial tumor offering easy access *via* the transdermal route. We initially studied the therapeutic effect of the MN platform by examining the ability of the CpG-NPs to instigate antitumor immunity when delivered with the MNs (**Figure 3A**). Five days-post tumor inoculation, B16-F10 tumor-bearing mice were administered with MNs loaded with CpG-NPs (CpG-NP MNs), MNs loaded with CpC_Ctrl_-NPs (CpC_Ctrl_-NP MNs) or empty MNs. The therapeutic regimen consisted of treatment every three days for five cycles (q3dx5). Treatment with CpG-NPs MNs resulted in a significant delay in tumor growth (**Figure 3B**) and a corresponding increase in the survival time compared to the control groups (CpC_Ctrl_-NP MNs and empty MNs) (**Figure 3C**). The therapeutic benefit of CpG-NP MNs, when delivered transdermally, was evidenced by the 3-fold lower tumor volume at the end of the study [≈ 800 mm^3^ in the control groups (**Figure 3D**) versus ≈ 200 mm^3^ in the treatment group (**Figure 3E**)]. In addition, we observed that 1 μg of CpG-NPs per mouse was well tolerated as no body weight loss was observed during the treatment period (**Figure S11**). In agreement with previous reports 36, the nanoencapsulation of the CpG induces therapeutic effects at a much lower dose than when delivered as a free drug intratumorally 46, validating the benefit of using NPs for nucleic acid delivery. The therapeutic merit of the MNs was demonstrated here in a superficial cancer model, yet we foresee the use of our MN platform to treat neoplasms in deeper tissues. Indeed, some authors have successfully reported the use of microneedles for the treatment of ovarian and triple negative breast cancer as well as metastatic ones, especially in the context of anti-cancer vaccines 4.

### MNs can release CpG-NPs in the TME for tumor suppression, as confirmed by IVM

**Figure 4:**
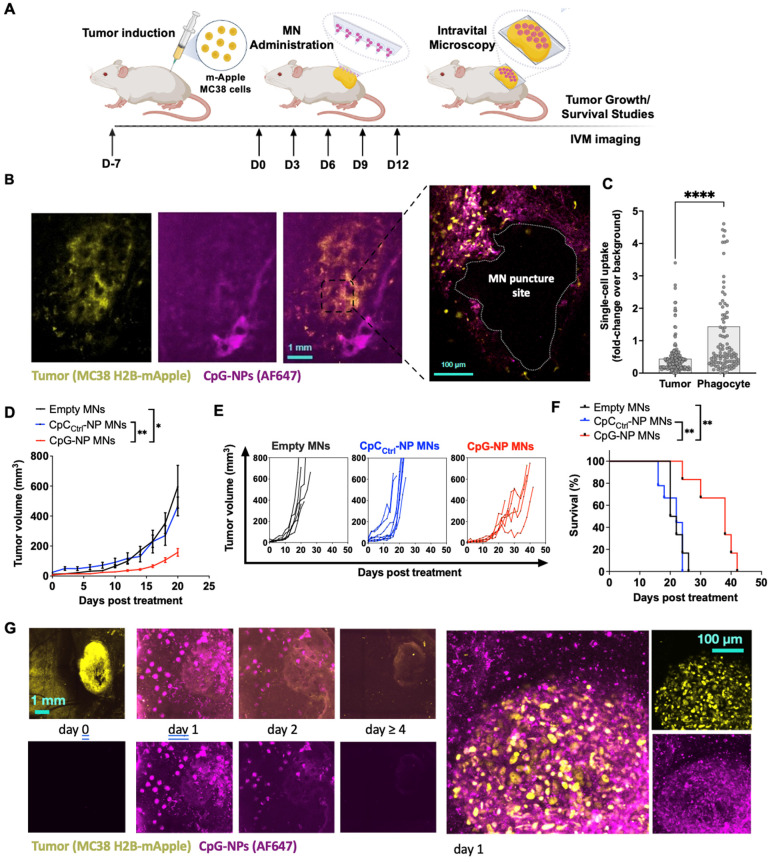
** Intravital microscopy (IVM) reveals the spatiotemporal localization of CpG-NPs in the TME following MN transdermal delivery, as well as their therapeutic efficiency. A,** Experimental design of the *in vivo* studies. **B,** IVM of mApple expressing MC38 tumors (yellow) treated with fluorescent CpG-NPs (magenta) loaded in the MNs. MNs were applied to the mApple MC38 tumors for 24 h and removed before IVM imaging. **C,** Single cell uptake quantification of CpG-NPs by IVM. Data are mean ± s.e.m. **D-F,** Mice with subcutaneous MC38-mApple tumors were treated seven days post-tumor induction with empty MNs, MNs loaded with CpC_Ctrl_-NPs (1 µg CpC-ODN delivered/patch) or MNs loaded with CpG-NPs (1 µg CpG-ODN delivered/patch) three days apart for five cycles (q3dx5), with tumor growth measured by caliper and shown as average volumes (**D**), individual growth curves (**E**), and Kaplan-Meier humane survival curves (**F**) (n = 8-10, data are mean ± s.e.m.). Statistical significance was determined between groups by the Mantel-Cox test. ***P < 0.001, **P < 0.01, *P < 0.05. **G,** mApple tumor fluorescence over time, measured by IVM (n = 2-5, data are mean ± s.e.m.). **H,** IVM of MC38-mApple tumors (yellow) treated with fluorescent CpG-NPs (magenta) (left, scale bar: 1 mm; right, scale bar: 100 µm).

After confirming the potential of the MNs in a mouse model of melanoma, we studied the spatiotemporal distribution of CpG-NPs in the TME when delivered using MNs by intravital microscopy (IVM). To do so, we used the syngeneic MC38 cancer model with growth characteristics and fluorescent protein expression ideal for intravital microscopy. MC38 tumor cells expressing H2B-mApple nuclear-localized fluorescent protein were implanted into the dorsal skinfold window chambers (DSWC) in C57BL/6J mice 47. MNs containing fluorescent AF647-labeled CpG-NPs were applied to tumor-bearing mice 7 days after tumor induction. After 24 h, the MN patch was removed for imaging (**Figure 4A**), which revealed a relatively homogeneous distribution of CpG-NPs from the patch into the neighboring tumor tissue and around the MN puncture sites (**Figure 4B**). Single-cell analysis of CpG-NP uptake indicated accumulation in H2B-mApple^+^ tumor cells and non-malignant phagocytic host cells consistent with known morphology and localization of tumor-associated macrophages in this model 47 (**Figure 4C**).

We next studied the therapeutic efficacy of transdermal delivery of CpG NPs using the subcutaneous MC38 colon cancer model. Tumors responded similarly to orthotopic B16-F10 tumors: treatment with CpG-NP MNs inhibited tumor growth (**Figure 4D-E**) and extended overall humane survival (**Figure 4F**). IVM also revealed a robust tumor response using the DSWC, where the loss of fluorescent signal reflected a loss of viable mApple^+^ tumor cells over time after a single CpG-NP MNs application (**Figure 4G**). Finally, NP tumoral distribution and release kinetics from the patch was investigated using IVM, showing the release of NPs into the tumor tissue over the 24 h application duration and extended NP retention within the tumor tissue — as seen at 48 h, when MN patches were removed for analysis (**Figure 4H**). In contrast, NPs injected intratumorally presented a more localized distribution (**Figure S12A**), and by 24 h post-injection, 95% of the NPs had cleared from the tumor tissue (**Figure S12B**).

### Analysis of the therapeutic potential of MNs for modulating the tumor microenvironment

**Figure 5:**
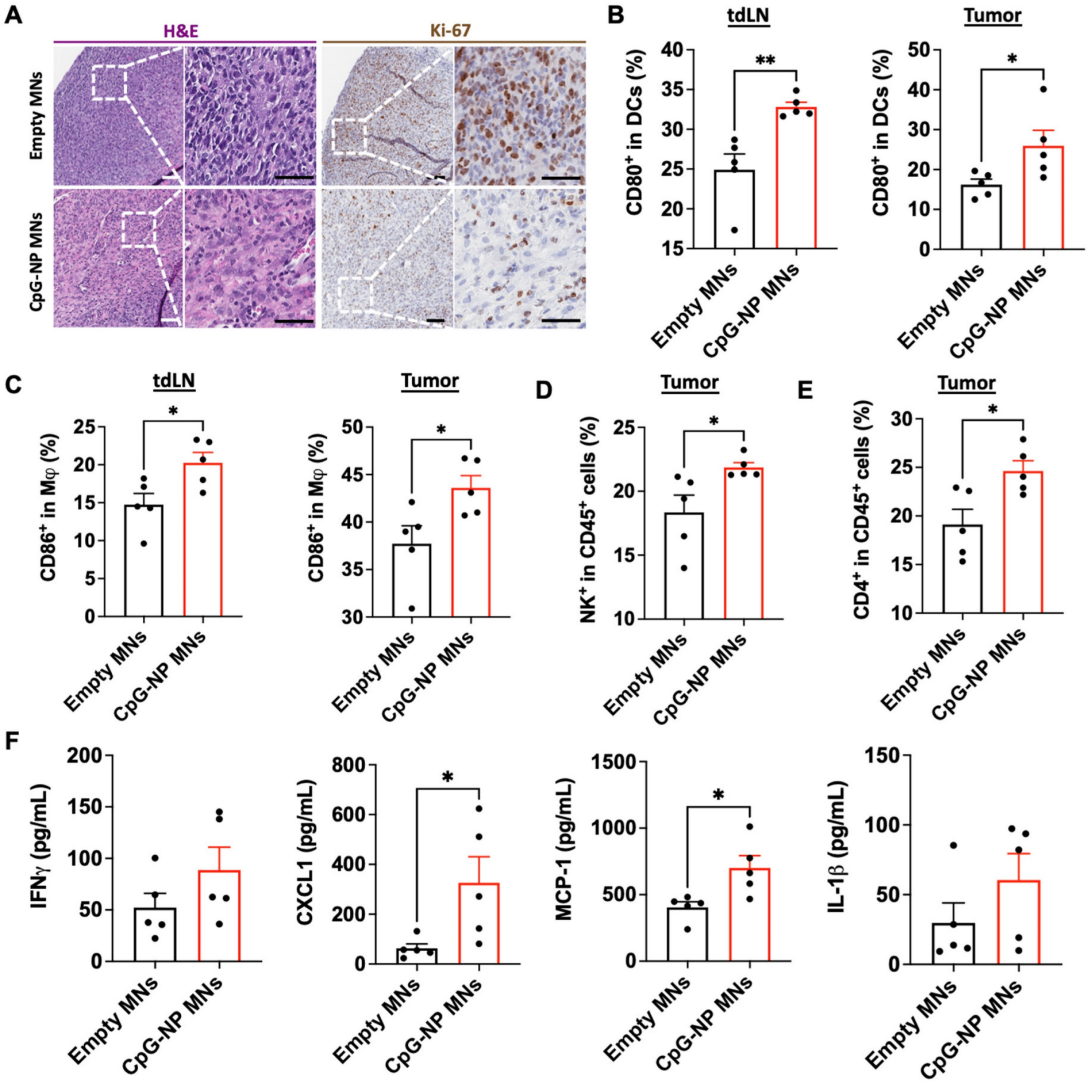
** Delivery of CpG-NPs using MNs modulates the immune composition in the TME. A,** Representative H&E staining (left, scale bar: 100 μm; right: scale bar: 50 μm) and Ki-67 staining (left, scale bar: 100 μm; right: scale bar: 50 μm) of MC38 tumors harvested on day 20 post-tumor induction (n = 5). **B,** Flow cytometry quantification of activated dendritic cells (CD80^hi^ CD11c^+^MHCII^+^CD45^+^) in tdLNs (left) and tumors (right) 48 h post transdermal delivery of CpG-NPs. Data are mean ± s.e.m. (n = 5). **C,** Flow cytometry quantification in the tdLNs (left) and tumors (right) of activated macrophages (CD86^hi^ F4/80^+^CD11b^+^CD45^+^) 48 h following MN-mediated delivery of CpG-NPs. Data are mean ± s.e.m. (n = 5).** D-E,** Flow cytometric quantification of the percentage of natural killer cells (d) and CD4^+^ cells (e) in tumor lysates 48 h post-treatment with CpG-NPs. **F,** Analysis of the pro-inflammatory cytokine/chemokine profile in tumors using a bead-based immunoassay (Legendplex™). Data are mean ± s.e.m. (n = 5). ***P < 0.001, **P < 0.01, *P < 0.05. Data were analyzed by Grubbs' test for statistical outliers, which were pre-defined using an alpha value of 0.01.

To understand the mechanisms by which the transdermal delivery of CpG-NPs resulted in enhanced antitumor activity, we performed immunohistochemical analysis of MC38 tumors following therapy (CpG-NP MNs, q3dx5). In agreement with the macroscopic findings, immunohistochemical staining for H&E and Ki-67 showed less actively proliferating cells in tumors treated with CpG-NP MNs than in the empty MNs control group (**Figure 5A**). In addition, we characterized the composition and the phenotype of the immune cell populations in the TME and tumor-draining lymph nodes (tdLNs) in MC38 tumor-bearing mice two days after a single administration of CpG-NP MNs, with a particular focus on early immune responders. Expression of CD80 by DCs (CD11c^+^ MHCII^+^) in the TME and tdLNs was significantly increased in the animals treated with CpG-NP loaded MNs (**Figure 5B**). A similar trend was observed for the macrophages (CD11b^+^ F4/80^+^), where an increase in CD86 expression was observed in treated mice compared to the control group receiving empty MNs (**Figure 5C**). These results support that CpG TLR-agonists are potent stimulators of DC and macrophage maturation and drive the expression of co-stimulatory receptors, including CD80 or CD86 48. Increased frequencies of natural killer (CD45^+^NK^+^) cells were observed in tumors administered CpG-NP MNs, in agreement with prior literature showing that TLR-agonists such as CpG motifs can induce NK cell lytic activity to support anti-tumor immunity 48,49 (**Figure 5D**). We also observed higher frequencies of CD4^+^ T cell infiltrates in the treated group compared to the control group (**Figure 5E**). Lastly, we examined how transdermal delivery of CpG-NPs affected downstream expression of proinflammatory cytokines and chemokines known to mediate innate and adaptive immune cell activation. Analysis of the tumor lysates confirmed that CpG-NP MNs increased the expression of interferon gamma (IFN-γ) in the TME compared to control mice receiving empty MNs. In addition, tumors treated with CpG-NP MNs showed increased expression of leukocyte chemokines, including the monocyte chemoattractant protein 1 (MCP-1) and the C-X-C motif chemokine ligand 1 (CXCL1), and pro-inflammatory cytokines such as interleukin 1 beta (IL-1β) responsible for immune cell recruitment 50 (**Figure 5F**). Overall, these results demonstrate the immunomodulatory potential of the MN platform, leading to a pro-inflammatory TME phenotype.

### Non-invasive cell sampling using HA-based MNs allows longitudinal monitoring of immunotherapy response

**Figure 6:**
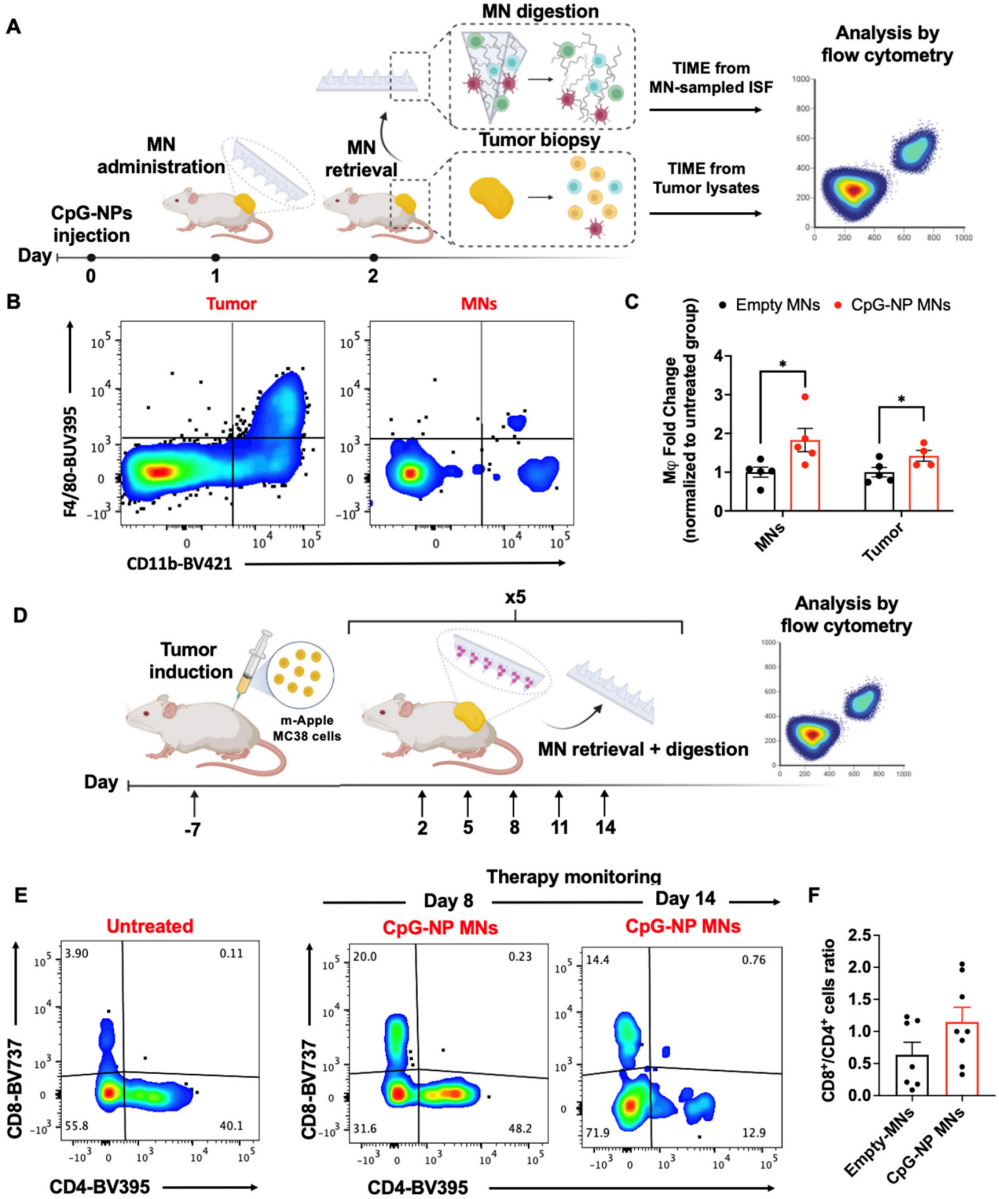
** Serial monitoring of the immune composition of the TME in ISF using HA-based MNs. A,** Scheme of MN immune cell sampling. After a single-dose injection of CpG-NPs, MNs were administered and digested with TCEP upon retrieval for subsequent analysis of the cellular immune signature compared to that from tumor lysates by flow cytometry. **B-C,** Flow cytometry dot plot (left) and quantification of macrophages (CD11b^+^ F4/80^+^) (right) recovered with MNs at day 2 following treatment. Data are mean ± s.e.m. (n = 4-5). ***P < 0.001, **P < 0.01, *P < 0.05. **D,** Serial monitoring of T cell infiltrates in ISF following treatment with CpG-NP MNs. **E,** Representative dot plot of CD8^+^ and CD4^+^ T cell populations over time from MN-sampled ISF. **F,** Ratio of CD8^+^ to CD4^+^ T cells extracted with MNs representing pooled values from day 11 and day 14 post-treatment initiation. Data are mean ± s.e.m. (n = 7-8). ***P < 0.001, **P < 0.01, *P < 0.05.

We next investigated the diagnostic capacity of the MN platform and its ability to extract ISF for minimally-invasive monitoring of the TME. We aimed to utilize the MN platform to longitudinally monitor the immune changes in the TME profile in ISF, which we hypothesized would shift from immunosuppressive to pro-inflammatory following TLR engagement. Following CpG-NPs therapy administration, empty MNs were applied for 24 h and digested to analyze proximal responders of TLR stimulation (**Figure 6A**, left). The entire MN matrix was composed of highly swellable and digestible HA hydrogel that allowed us to capture and analyze roughly 1,000 CD45^+^ cells per patch in a theoretical volume of 3 mm^3^ (**Figure S13-16**). Next, we were able to further gate this subset cellular population by flow cytometry to examine the cellular frequencies of the main contributors to anti-tumor immunity, such as macrophages, and compare them with those found in tumor lysates (**Figure 6B, right**). Flow cytometric quantification of the cellular suspensions recovered from MNs on day two post-treatment confirmed an increased percentage of macrophages (CD11b^+^ F4/80^+^ CD45^+^) in the MN-recovered ISF from treated mice compared to that of the control group (**Figure 6B-C**). The same trend was observed in the corresponding tumors, which registered increased frequencies of macrophages in mice administered with CpG-NPs (**Figure 6B-C**), supporting a correlation between cells extracted from MN patches and the bulk TME in response to the immunotherapy.

In a second study, we focused on monitoring the adaptive immune responses that may evolve more gradually, particularly examining effector and helper T cells. Here, CpG-NP MNs were serially administered following the same therapeutic regimen as for the efficacy studies (q3dx5). Cellular changes in the MN-sampled ISF were analyzed by flow cytometry (**Figure 6D**). Mainly, we focused on the population of tumor-infiltrating lymphocytes, using the CD8^+^/CD4^+^ T cell ratio as a prognostic biomarker of responsiveness to immunotherapy 51-53. Analysis of the MN-sampled TME infiltrate revealed an increase in the CD8^+^ T cell population in treated mice compared to the control group, which was evident in all the samples analyzed over time (**Figure 6E**). Also, an increase in the CD8^+^/CD4^+^ ratio following immunotherapy was observed, although not statistically significant, compared to untreated mice (pooled frequencies from day 11 and day 14 post-treatment initiation) (**Figure 6F**). Here, the correlation between the T cell signature in the TME and the response to therapy could only be established qualitatively due to the low frequencies of effector T cells inherent to the MC38 model 54. Despite the experimental constraints —which we hypothesize could be avoided if profiling T-cell-inflamed tumors— we confirmed the potential of the diagnostic arm of the MN platform as it allowed identifying cellular subsets such as effector T cells that were scarce in the TME. To our knowledge, correlating the TME composition by MN analysis with the bulk tissue state of the tumor has only previously been pursued with soluble biomarkers (such as proteins and nucleic acids 19-21). Here, we describe the use of cellular biomarkers sampled from ISF to infer the state of the tumor and enable prognosis following treatment by minimally invasive means. These results support the potential use of our HA-based MNs to sample the immune cell profile in the TME. The correlation between the immune signature of MN-extracted cells and that of the bulk tumors for DC, macrophage, and T cell populations supports the potential of this platform for future theranostic avenues.

## Conclusions

Our dual-function MN platform enables minimally invasive immunotherapy administration and analytical sampling of biomarkers in the context of cancer management. The hydrogel matrix of the MNs was loaded with NPs encapsulating an immunomodulatory agent (CpG-ODNs), enabling their localization at the tumor site, as observed by IVM. Following MN administration, the tumor burden was reduced when treating an orthotopic melanoma cancer model and a subcutaneous colorectal cancer model. Mechanistic studies confirmed the activation of DCs and macrophages in response to TLR engagement and the remodeling of the TME towards an immune-stimulatory state, which was evidenced by the increased levels of pro-inflammatory cytokines and chemokines in tumor lysates. MNs facilitated longitudinal monitoring of the changes in the TME, focusing on the innate arm of the immune system at early time points followed by monitoring of the adaptive responses. We reported an increase in macrophages in MN-sampled ISF after treatment that correlated with the immune profile in tumors, as well as an average increase in the infiltrates of effector and helper T cells. We foresee that integrating therapeutic and diagnostic functionalities of hydrogel-based MNs in a single, theranostic patch will open up prospective clinical avenues for enhanced patient management, offering minimally invasive intervention and allowing for multiplex analysis of cellular biomarkers involved in anti-tumor immunity.

## Materials and Methods

### Materials

All reagents and solvents were purchased from Sigma Aldrich unless otherwise stated. Sodium hyaluronate (60 kDa) was obtained from LifeCore Medical with a purity of at least 95%. NHS-terminated 8-arm PEG was purchased from Creative PEG Works. Microneedle PDMS custom-made molds (11 X 11 needles with a height 600 µm, base width of 300 µm, and tip to tip spacing of 600 µm) were obtained from Blueacre Technology. Arginine peptide (H-Cys-Arg-Arg-Arg-NH2) was obtained from CPC Scientific with a purity of at least 90%. ODN 1826 (CpG) and control ODN 1826 were purchased from InvivoGen.

### Synthesis of pBAE polymers functionalized with arginine polypeptide

Polymers were synthesized in accordance with previous work 30. PBAE polymerization was performed by mixing 5-amino-1-pentanol (0.426 g, 4.1 mmol), hexylamine (0.422 g, 4.1 mmol), and 1,4-butanediol diacrylate (2.0 g, 9.1 mmol) under magnetic stirring at 90 °C for 24 h. Next, acrylate moieties were end-capped with thiol-terminated arginine peptide at 1:2.1 pBAE:peptide molar ratio in dimethyl sulfoxide (DMSO). The mixture was stirred overnight at room temperature, and the resulting polymer was precipitated with a mixture of diethyl ether and acetone (70:30 v/v). Arginine modified pBAE polymer (C6-CR3) structure was confirmed by 1H-NMR (400 MHz Varian NMR spectrometer). To synthesize the fluorescent pBAE polymer the acrylate moieties were end-capped with amine-terminated AF647 dye at 1:2.1 pBAE:AF647 molar ratio in dimethyl sulfoxide (DMSO). The mixture was stirred overnight at room temperature, and the resulting polymer was precipitated as previously has been described.

### CpG retardation assay

To assess CpG complexation, different CpG to C6-CR3 ratios (w/w) (between 1:1 and 400:1) were studied. CpG-NP were freshly prepared and loaded in 4% E-Gel Precast Agarose Gels (Thermo Fisher), run following the manufacturer's instructions, and visualized in fluorescence mode.

### Formation of NPs

NPs were generated by mixing equal volumes of CpG-ODN 1826 at 0.4 mg/mL and C6-CR3 polymer at 20 mg/mL in sodium acetate buffer (AcONa) at 12.5 mM, followed by 5 min incubation at room temperature (RT). Next, this mixture was nano precipitated with 2.5 volumes of PBS and incubated for 20 min at RT to form NPs. The final CpG-NPs were concentrated by centrifugal filtration (10kDa MWCO) and sterilized by filtration (0.22 μm). Similarly, CpCCtrl-NPs were formulated following the same procedure but using a CpC-ODN 1826 control.

### Biophysical characterization of NP

Dynamic light scattering (DLS) was performed to determine the size and surface charge of the NPs. Briefly, 100 μL of CpG-NP or CpCCtrl-NPs were diluted with 900 μL of PBS and analyzed using a Zetasizer Nano ZS equipped with a He-Ne laser (λ = 633 nm) at a scattering angle of 137° (Malvern Instruments Ltd, United Kingdom). NP size was also analyzed by transmission electron microscopy (TEM) by the microscopy core at the Koch Institute for Integrative Cancer Research.

### Synthesis of Amino-modified hyaluronic acid (HA-SS-NH_2_) polymer

The HA-SS-NH_2_ polymer was synthesized following a previously described procedure 25. Briefly, 60 kDa-sodium hyaluronate (1% w/v in MES buffer) was activated with N-(3-(dimethylamino)propyl)carbodiimide (EDC) and N-hydroxysuccinimide (NHS) at a 1:4:2 molar ratio and reacted at room temperature for 30 min. The activated hyaluronic acid (HA) was then mixed with cysteamine dihydrochloride at a 1:10 molar ratio and reacted at room temperature for 12 h. HA-SS-NH_2_ was purified by dialysis, freeze-dried, and stored at -20° C. HA-SS-NH_2_ molecular structure was characterized by ^1^H-NMR using D_2_O as a solvent (400 MHz Varian NMR spectrometer).

### HA-based MN fabrication

MNs were fabricated as described in our previous work 25, using custom-made molds (11 x 11 array of negative MNs projections, each with a height of 600 µm and a radius of 150 µm). First, HA-SS-NH2 polymer (10% w/v in phosphate buffer, pH = 7.4) was cast on top of the molds by centrifugation at 4200 rpm. The excess polymer was carefully removed, and molds were freeze-dried. Then, an 8-arm-PEG-NHS crosslinker (10% w/v in phosphate buffer, pH = 7.4) was added to the HA-SS-NH2 polymer and cast by centrifugation under the same conditions. Next, a solution containing CpG-NPs and glycine (10 ng/mL) was deposited carefully on top of the projections area of the mold, put in a vacuum for 2 min, and centrifuged for 1 min. Immediately after, a polymeric backing layer of PLGA [Resomer® RG 505, Poly(D,L-lactide-co-glycolide), Sigma-Aldrich, USA] at 15% (w/v) dissolved in acetonitrile was added dropwise until covering the whole area of the mold. Finally, CpG-NP loaded MNs were dried at room temperature for 12 h, peeled off the molds carefully, and stored at room temperature.

### Cell Lines

*Mus musculus* skin melanoma (B16-F10 from ATCC) and colon carcinoma (H2B-mApple MC38, developed and described previously 47, were maintained in Dulbecco's minimum essential medium (DMEM) supplemented with 10% (v/v) fetal bovine serum (FBS), 100 U/mL penicillin and 100 μg/mL streptomycin, 2 mM L-glutamine. HEK-Blue mTLR9 cells (InvivoGen) were similarly maintained with the addition of 100 μg/ml of Normocin^TM^ and Zeocin^TM^ and 30 μg/ml of blasticidin. B.

### Bone marrow-derived dendritic cell (BMDC) and bone marrow-derived macrophage (BMDM) isolation

The tibias and femurs of female C57BL/6 mice (aged 8-12 weeks) were isolated and flushed to harvest bone marrow and obtain a progenitor cell population. To generate BMDMs, 4-6×10^6^ bone marrow cells were cultured in non-tissue culture-treated T175 flasks with DMEM/F12 supplemented with 10% (v/v) FBS, 1% (v/v) P/S, 5% (v/v) GlutaMAX and recombinant murine macrophage-colony-stimulating factor (M-CSF, 20 ng/mL). The flasks were supplemented with the additional medium on day three and day six. BMDMs phenotype was confirmed as CD11b^+^ F4/80^+^ by flow cytometry. To generate BMDCs, 2x10^6^ bone marrow cells were added to non-tissue culture-treated Petri dishes and cultured in 10 mL of RPMI-1640 supplemented with 10% (v/v) FBS, 1% (v/v) P/S and recombinant murine granulocyte-macrophage colony-stimulating factor (GM-CSF, 20 ng/mL, Biolegend). Plates were replenished with fresh, supplemented media on day three, six and every other day after that until use. Cell harvesting was performed as follows: BMDMs were detached using Accumax^TM^ (Sigma), and BMDCs were loosely adherent and could be collected by simple washing. BMDCs phenotype was confirmed as CD11c^+^ MHCII^+^ by flow cytometry.

### *In vitro* evaluation of CpG-NP activity

HEK-Blue mTLR9 cells were seeded in 96-well plates at 2 × 10^5^ cells per well and incubated with the CpG-NP, CpC_Ctrl_-NP, free CpG, or free CpC_Ctrl_ at concentrations ranging from 0 nM to 6 nM. At 24 h post-treatment, NF-κB activity was determined using the HEK-Blue Detection^TM^ reagent (InvivoGen) according to the manufacturer's instructions.

### Cell viability studies

HEK-Blue mTLR9 cells were seeded in 96-well plates at 2 × 10^4^ cells per well. Cells were treated with different concentrations of CpG-NPs and CpC_Ctrl_-NP for 24h, and cell viability was assessed using the MTS assay (Promega) as instructed by the manufacturer. Cells were incubated for up to 2 h, and absorbance was measured at 490 nm using a plate reader.

### Cell internalization studies

HEK-Blue mTLR9 cells, BMDCs, BMDMs were seeded in 24-well plates at 1 × 10^5^ cells per well and incubated with fluorescently labeled CpG-NP at CpG concentrations ranging from 0.0001 to 100 nM. After 4 h, CpG-NPs were removed, cells were washed, trypsinized, and fixed with 4% (v/v) paraformaldehyde for 10 min. CpG-NPs internalization was determined by flow cytometry.

### Primary immune cell activation using CpG-NPs

BMDCs or BMDMs were seeded in 24-well plates at 1 × 10^5^ cells per well and incubated with CpG-NP or CpCCtrl-NP at 10nM. After 24 h, the supernatant was removed, and cells were then collected and analyzed by flow cytometry. The following antibodies were used: CD11c BV421 (clone N418), MHCII BV605 (clone M5/114.15.2), CD80 FITC (clone 16-10A1), CD86 PE (clone GL-1), F4/80 BUV395 (clone T45-2342), CD11b BV421 (clone M1/70), CD86 FITC(clone GL-1), CD80 APC (clone 16-10A1), CD206 PE (clone C068C2). Live cells were gated using LIVE/DEADTM (Thermo Fisher) near-IR (cat. no. L34976). In addition, CCL2 (MCP-1), CXCL1 (KC), CXCL10 (IP-10)), TNF-α and IL10 were analyzed from the BMDM supernatant using a custom LegendplexTM panel (BioLegend) as per the manufacturer's instructions.

### Analysis of the mechanical properties of the HA-derived MNs

Mechanical strength of the MNs when empty or NP-loaded were measured using a micro-force test station with a mechanical sensor (3400 Series, Instron, USA). MN patches were placed on the surface of the platform with the needle-like projections facing up. The displacement and force applied to the MNs were recorded from the moment the sensors touched the uppermost tip of the MNs until a maximum force of ≈100 N was reached. Force-travel curves of MNs arrays were obtained by correlating the compressive strain or displacement (%) with the compressive stress (kPa), and Young's modulus was determined from the slope (GPa). Swelling ability of the MNs was evaluated *in vitro* by incubating the MNs in PBS for 24 h and recording the weights upon recovery. Swelling percentage was obtained using the following equation where: W(i.): MN weight following incubation and AW0: Average of MNs weights prior incubation.



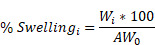



### Study of CpG-NP release kinetics from the MNs *in vitro*

To assess the release of CpG-NPs from the HA MNs, CpG-NPs were conjugated with Alexa Fluor 647 before the microneedle fabrication. CpG-NP-loaded MNs were placed in Eppendorf tubes, immersed with PBS (1 mL), and incubated under rotation at 37 °C. 100 µL of releasing media was replaced at a predetermined time point, and fluorescence of the release NPs was measured by microplate reader [Infinite M Plex multimodal plate reader (TECAN)] (λ_ex_ = 640 nm, λ_em_ = 680 nm).

### On-demand digestion of HA-derived MNs

HA-based MNs from *in vitro* studies or retrieved from mice were incubated with 10 mM Tris (2-carboxyethyl) phosphine (TCEP) solution in PBS at pH 7.4 with rotation at 37 °C for 5 min. The recovered cellular suspension was filtered with a 70 µm cell strainer (BD Biosciences) to remove any impurities and further processed by flow cytometry. Digestion of the MNs following incubation was confirmed by fluorescence microscopy using fluorescently-labeled MNs. To confirm on-demand digestibility, fluorescently-labeled MNs fabricated with AF647-conjugated HA were incubated with either the TCEP solution or PBS as control. Next, the supernatants were collected, and the levels of fluorescence were analyzed in a microplate reader (λ_ex_ = 640 nm, λ_em_ = 680 nm).

### Analysis MN loading capacity

First, MNs were loaded with a solution of fluorescently-labeled NPs on top of the mold as described here. After drying and demolding, the resulting MNs were digested by incubating them in digestion buffer (10 mM TCEP, PBS, pH = 7.4) and the fluorescence of the digested MNs was quantified in a microplate reader ((λ_ex_ = 640 nm, λ_em_ = 680 nm). Finally, fluorescence measurements were compared to the theoretical ones and the percentage of total loading capacity was obtained.

### Analysis of CpG-NPs release kinetics from the MNs *in vivo*

HA-MNs were loaded with CpG-NPs labeled with Alexa Fluor 647 (2 µg of CpG/MN patch) and subsequently applied on the back of healthy C57BL/6 mice (2 MN patches/mice) for increasing durations of time (t = 3 h, t = 6 h and t = 24 h). Next, MNs were recovered and digested as described herein. Fluorescence of the resulting MN suspensions (associated with the remaining NPs still in the MN matrix) was analyzed in a microplate reader (λ_ex_ = 640 nm, λ_em_ = 680 nm). Release profile was obtained by subtracting the fluorescence levels from those registered from control MNs that had not been applied.

### Animal Experiments

Female C57BL/6 mice (6-8 weeks old) were purchased from Charles River. Mouse procedures were conducted at the Koch Institute for Integrative Cancer Research at the Massachusetts Institute of Technology (MIT) under the protocol approved for this study by the Institutional Animal Care and Use Committee (IACUC). Intravital microscopy studies were performed at the Massachusetts General Hospital under a protocol approved for this study by the Institutional Animal Care and Use Committee (IACUC), using female C57BL/6J mice (8-12 weeks old) provided by Jackson Labs.

### *In vivo* therapeutic efficacy and survival studies

Two different tumor models were used. For mApple-expressing MC38 cells, 1×10^6^ cells in 100 μL of HBSS were injected subcutaneously into the right flank of the mice. For orthotopic B16-F10 cells, 5×10^5^ cells in 50 μL of HBSS were injected intradermally into the right flank of the mice. Five days post-tumor induction, mice were administered empty MNs (negative control), CpC_Ctrl_-NP-loaded MNs (negative control) or CpG-NP-loaded MNs (treatment group) 5 times, three days apart (n = 10 mice per group). MNs were administered on top of the tumor by thumb-pressing them against the skin or in the surrounding areas and secured with medical-grade tape (FlexCon, USA). MNs were retrieved 24 h post-administration. Tumor size was measured every other day via caliper measurements (n = 6), and the tumor volume was calculated using the equation V = (Length × Width × Height)/π ÷ 6. Body weight was measured contemporaneously with tumor volume. Mice were euthanized when tumors reached a volume of 1000 mm^3^ or when poor body condition was observed.

### Immunophenotyping analysis in the TME

Subcutaneous MC38 tumors were established in female C57BL/6 mice (6-8 weeks old) as previously described and two studies were conducted. Mechanistic analyses were performed after mice were administered CpG-NP loaded MNs (1 µg delivered per patch) or empty MNs either once or five times (day two and day 20-post treatment initiation, respectively) depending on the cellular populations of interest. To test the diagnostic potential of the MNs, a single dose of CpG-NPs (1 µg in 25 µL of PBS) was administered intratumorally. On the day of mechanistic analysis, tumors were harvested, chopped into < 0.5 mm fragments, and digested in a solution of HBSS supplemented with collagenase I, II, and IV (100 ng/mL), and DNase I (100 μg/mL) for 2 h at 37 °C. tdLNs were harvested and mechanically dissociated. Single-cell suspensions of tumors and tdLNs were filtered through a 40 μm nylon cell strainer. Tumor cells were further treated with ACK Lysing Buffer (Gibco) for 1 min. Cells were washed, filtered through a 40 μm nylon cell strainer, and counted. MNs recovered from mice were digested as described before. The following anti-mouse antibodies were used for flow cytometry were purchased from BioLegend: CD45 APC-Cy7 (clone 30-F11), NK-1.1 BV711 (clone PK136), CD45 BV785 (clone 30-F11), CD11b BV421 (clone M1/70), CD86 BV510 (clone GL-1), CD80 BV711 (clone 16-10A1), CD206 PE (clone C068C2), MHCII BV605 (clone M5/114.15.2), CD11c APC (clone N418). The following anti-mouse antibodies were purchased from BD Biosciences: CD3 BB700 (clone 17A2), CD4 BUV395 (clone GK1.5), CD8a BUV737 (clone 53-6.7), F4/80 BUV395 (clone T45-2342), CD80 BUV737 (clone 16-10A1). Live cells were gated using LIVE/DEADTM (Thermo Fisher) aqua (cat. no. L34966), green (cat. no. L34970), or near-IR (cat. no. L34976). Stained cells were analyzed by flow cytometry using a BD LSRFortessaTM flow cytometer (BD Biosciences), and all data were analyzed using FlowJo software (Flowjo LLC). Samples yielding low cell counts due to experimental constraints were removed prior to the analysis.

### Cytokine analysis

Half of the harvested tumors for immunophenotyping on day two were processed to analyze the cytokine/chemokines expression profile. Tissues were homogenized with T-PER™ Tissue Protein Extraction Reagent (Thermo Fisher Scientific, cat. no. 78510) containing 1% Halt protease and phosphatase inhibitors (Thermo Fisher Scientific, cat. no. 78442). Then, the samples were incubated at 4 °C for 30 min following centrifugation to remove the cell/tissue debris. The supernatant was collected for total protein quantification and cytokine analysis. Levels of IFNγ, CCL2 (MCP-1), CXCL1 (KC), and IL-1β were analyzed using a custom Legendplex™ panel (BioLegend) as per the manufacturer's instructions. Samples rendering bulk protein concentrations below the limit of detection by micro-BCA assay were not considered for analysis.

### Analysis of tumor proliferation by immunohistochemistry

Skin tissue sections were processed and imaged by the Hope Babette Tang Histology facility at the Koch Institute of Integrative Cancer Research at MIT (Cambridge, USA). Briefly, 1 cm^3^ tumor sections were harvested on the day of mechanistic analysis and were embedded in O.C.T. in plastic base molds for tissue embedding. Samples were flash-frozen and preserved at -80 °C until sectioning. Tumors were cryosectioned into 20 μm-wide tissue sections, and proliferation was assessed via H&E and Ki-67 staining. The microscopic images were processed using the Aperio ImageScope 12.3.3 software (Leica).

### Intravital microscopy

*In vivo* microscopy was performed using an Olympus FV1000 multiphoton/confocal imaging system following procedures described previously 47,55. Animals were used with Institutional Subcommittee on Research Animal Care guidelines. 5 × 10^5^ MC38-H2B-mApple cells in 50 μL PBS were injected under the fascia two days after DSWC implantation surgery and imaged ~7 days later upon tumor formation. MN patches were applied onto the tissue within the window chamber, and glass coverslips were replaced over the patch to protect the tissue. 24 h later, patches were removed, and tissue was imaged after adding saline and replacing the coverslip. An XLFluor X2 air objective (numerical aperture 0.14, Olympus) was used for low-magnification imaging of the entire tumor and MN delivery region. In contrast, higher-magnification imaging was conducted with an XLUMPLFLN ×20 water immersion objective (numerical aperture, 1.0, Olympus). Sequentially scanned images were acquired with 559- and 633-nm diode lasers and a DM405/488/559/635-nm dichroic beam splitter 42,50. Empty patches lacking both CpG and dye were re-applied under coverslips for 24 h repeated twice to mirror the serial treatment scheme in the subcutaneous experiments. Longitudinal imaging was performed for up to 9 days post-treatment.

### Statistical Analysis

Statistical analyses were performed using Graph-Pad Prism 8 (GraphPad Software). For *in vitro* experiments, a minimum of n = 3 biological replicates were used per condition in each experiment. The unpaired Student's t-test evaluated the statistical difference between the two measurements. A Post hoc test for one-way ANOVA was used to test the statistical difference between sets of measurements. For *in vivo* experiments, a minimum of n=4 biological replicates were used per condition in each experiment. Survival was estimated using Kaplan-Meier methodology and comparisons were made using the log-rank test. No specific pre-processing of data was performed before statistical analyses. Data were analyzed by Grubbs' test for statistical outliers, which were pre-defined using an alpha value of 0.01. Differences between groups were considered significant at p-values below 0.05 (* p < 0.05, ** p < 0.01, *** p < 0.001).
