# Supplementary material for: Polymeric microneedles enable simultaneous delivery of cancer immunomodulatory drugs and detection of skin biomarkers
Source: Theranostics. 2023 Jan 1;13(1):1–15. doi: 10.7150/thno.73966 (PMC9800729; doi:10.7150/thno.73966)
Supplement: Supplementary file 1 — Supplementary figures and tables. [file thnov13p0001s1.pdf]

Polymeric microneedles enable simultaneous delivery of cancer immunomodulatory drugs and detection of skin biomarkers

Pere Dosta[1,2,6]*, Núria Puigmal[1,2,6]*, Alexander M. Cryer[1,2,6], Alma L. Rodríguez[1,2], Ella Scott[3], Ralph Weissleder[3,4,5], Miles A. Miller[3,4], Natalie Artzi[1,2,6]

[1]Institute for Medical Engineering and Science
Massachusetts Institute of Technology
Cambridge, MA 02139

[2]Department of Medicine
Division of Engineering in Medicine
Brigham and Women's Hospital
Harvard Medical School
Boston, MA 02115
*E-mail: nartzi@mit.edu; nartzi@bwh.harvard.edu

[3]Center for Systems Biology
Massachusetts General Hospital Research Institute
Boston, MA 02114
*E-mail: miles.miller@mgh.harvard.edu

[4]Department of Radiology
Massachusetts General Hospital
Harvard Medical School
Boston, MA 02114

[5]Department of Systems Biology
Harvard Medical School

[6]Wyss Institute for Biologically Inspired Engineering
Harvard University
Boston, MA, 02115

Keywords: microneedles, immunotherapy, cancer, poly(beta-amino ester)s, theranostics

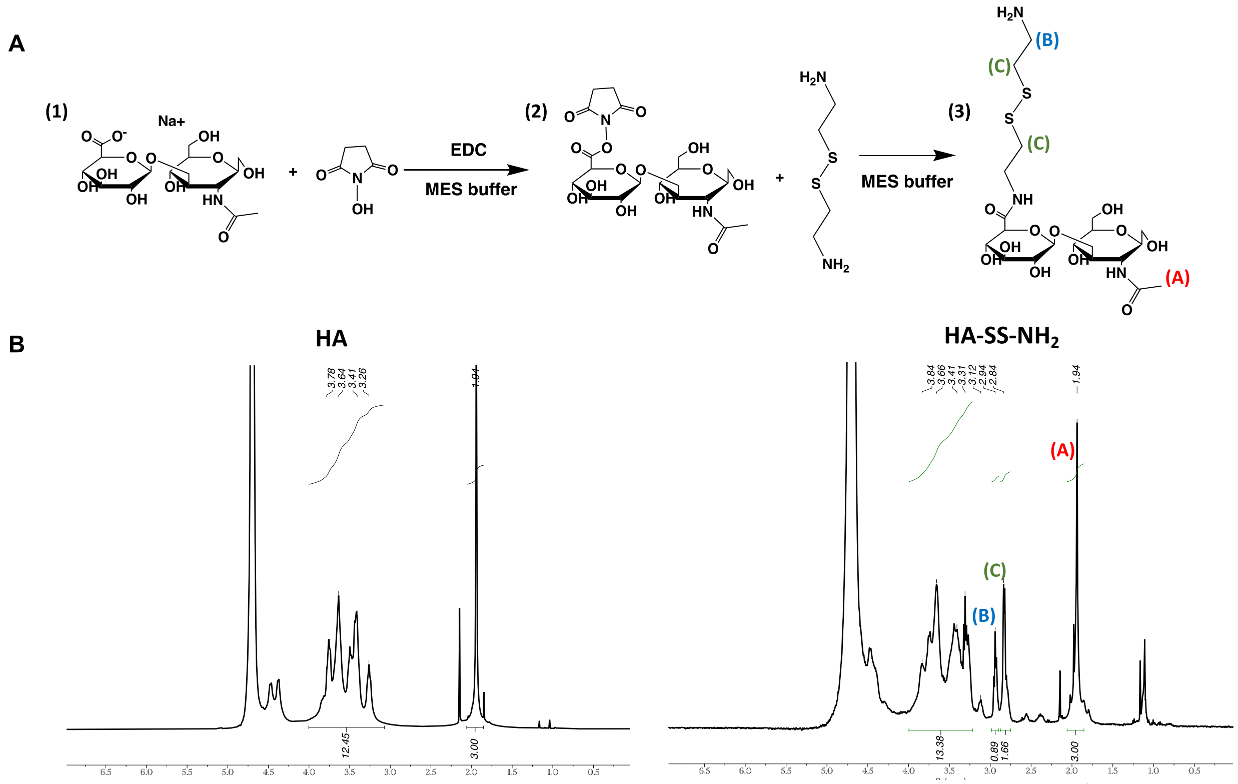

Figure S1: A, Synthesis, and chemical structure of HA-SS-NH_2 polymer. **B**, ^{1}H-NMR of unmodified HA and HA-SS-NH_2 polymer (400MHz, DMSO-*d6*, TMS) (ppm)

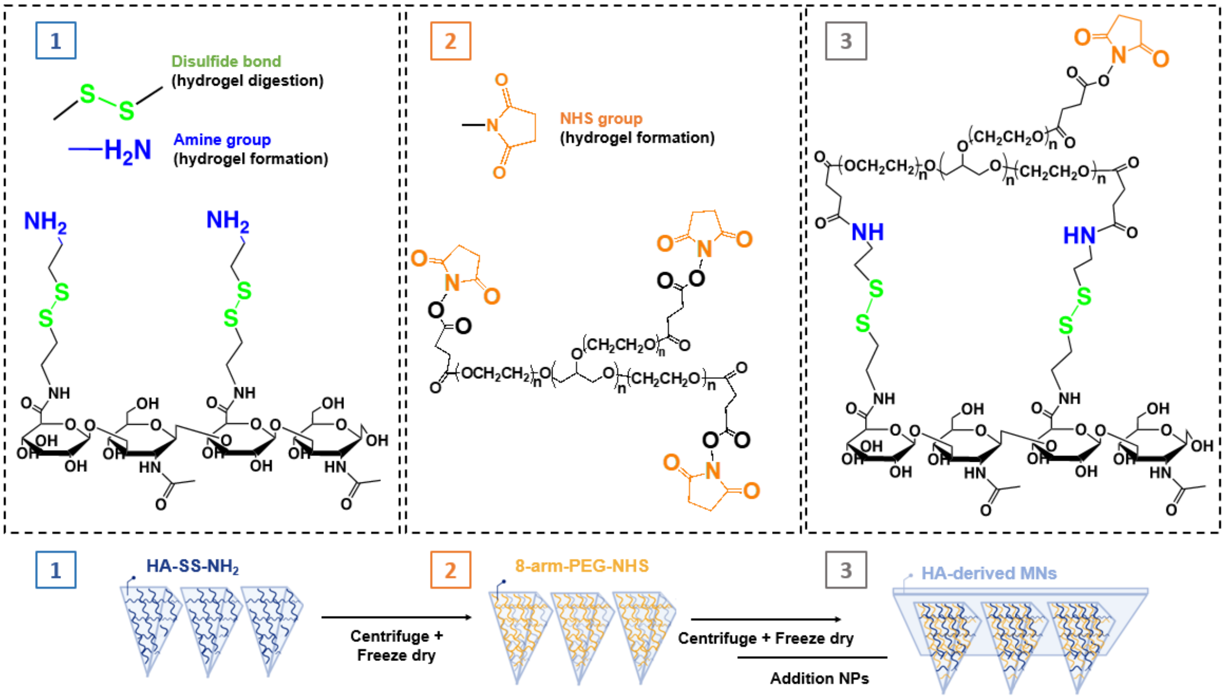

Figure S2: HA-based MN platform for simultaneous transdermal drug delivery and Interstitial fluid Sampling for cancer treatment. HA-based MN fabrication was performed by casting an aqueous amine-modified HA (HA-SS-NH_2) solution into the PDMS mold by centrifugation and crosslinked using the NHS-terminated 8-arm PEG crosslinker. CpG Nanoparticles were loaded and a PLGA back layer was added (bottom scheme).

a

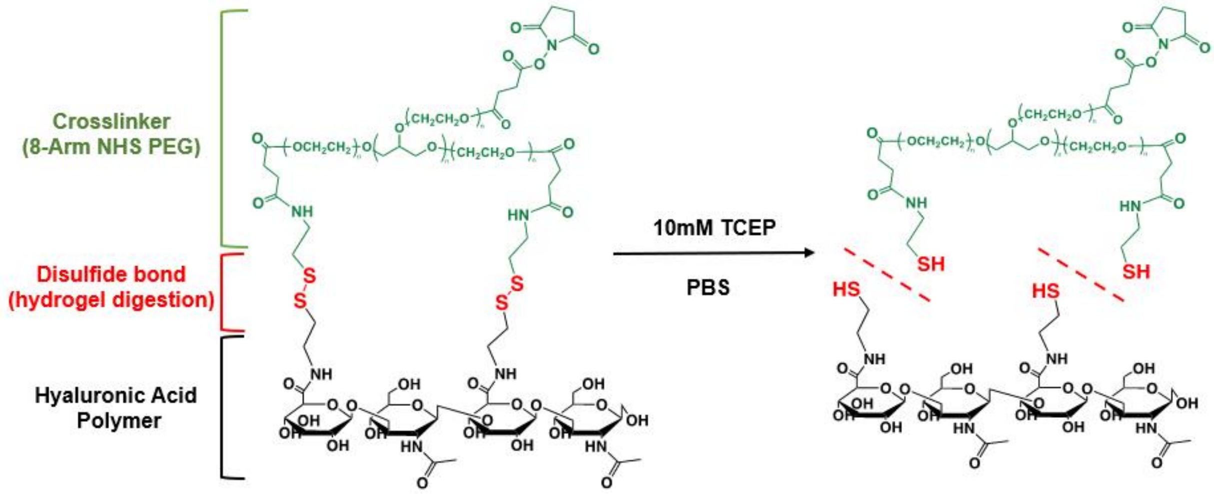

b

[TCEP] mM	Digestion time (min)
100	<5
10	<5
1	>60
0	-

c

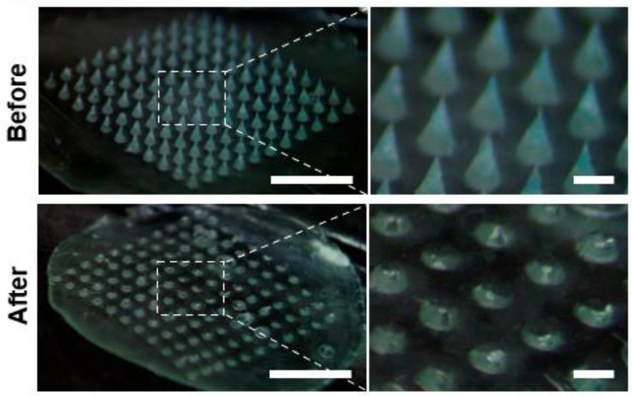

d

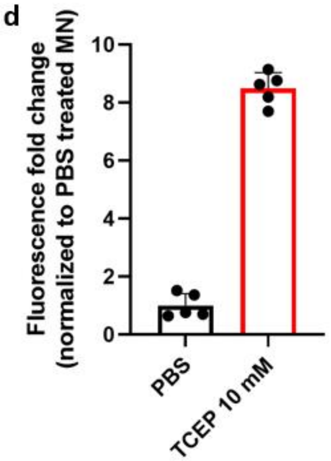

Figure S3: On-demand degradation of the MNs under reducing conditions for subsequent ISF recovery. A, Chemical structure of the HA-SS-NH_2 modified with a disulfide bond for on-demand degradation when incubated with the reducing agent TCEP. **B,** Digestion times of MNs using varying concentrations of the reducing agent TCEP. **C,** Microscopy images of the hydrogel-based MNs before (top) and after (bottom) incubation for 5 minutes with a 10 mM TCEP solution (Scale bar left = 2 mm, right = 300 µm). **D,** Fluorescence evaluation of AF647-conjugated HA that has been released into the supernatant after incubation of MNs with PBS (negative control) or on-demand digestion with TCEP (10 mM TCEP) for 5 min (n = 5).

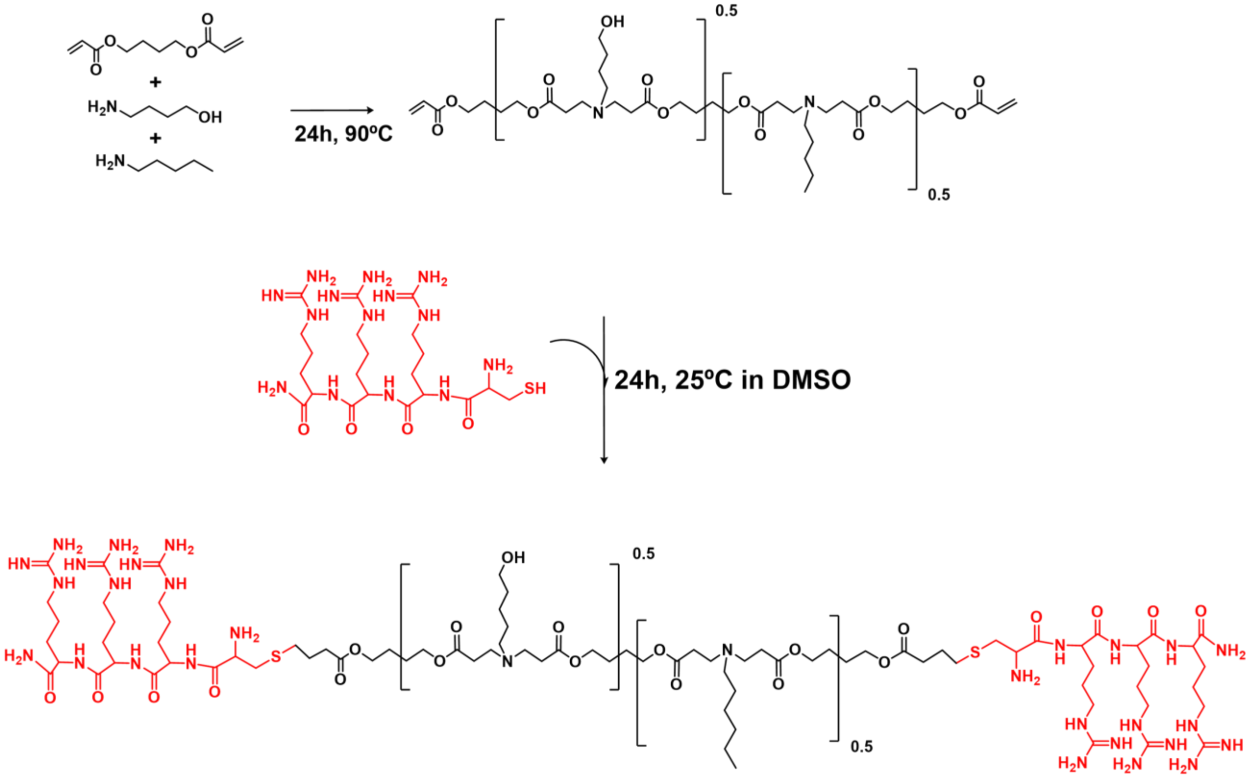

Figure S4: Synthesis of arginine modified poly (beta-amino) formulation. A, Synthesis of pBAE polymer. A mixture of 5-amino-1-pentanol, hexylamine, and 1,4-butanediol diacrylate (0.5:0.5:1.2) were used for the synthesis of pBAE C6 polymer. **B,** Arginine-modified pBAE are formulated mixing acrylate-terminate pBAE polymer with polyarginine peptide containing a cysteine amino acid (Cys-Arg-Arg-Arg).

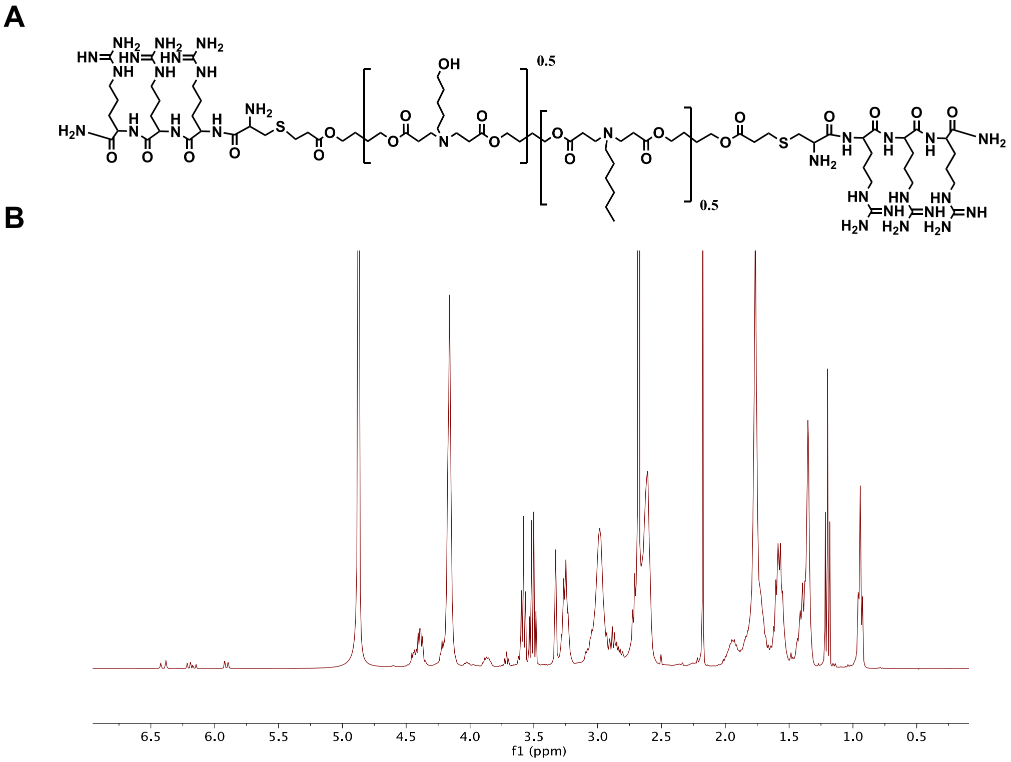

Figure S5: A, Chemical structure of C6-CR3 Polymer. **B,** ^{1}H-NMR of C6-CR3 Polymer (400MHz, Methanol-d_4, TMS) (ppm): δ = 4.41-4.33 (br, NH_2-C(=O)-C<u>H</u>-NH-C(=O)-C<u>H</u>-NH-C(=O)-C<u>H</u>-NH-C(=O)-C<u>H</u>-CH_2-, 4.16 (t, CH_2-C<u>H</u>$_2$-O), 3.58 (t, CH_2-C<u>H</u>$_2$-OH), 3.25 (br, NH_2-C(=NH)-NH-C<u>H</u>$_2$-, OH-$(CH_2)_4$-C<u>H</u>$_2$-N-), 3.04 (t, CH_2-C<u>H</u>$_2$-N-), 2.82 (dd, -C<u>H</u>$_2$-S-C<u>H</u>$_2$), 2.48 (br, -N-CH_2-C<u>H</u>$_2$-C(=O)-O), 1.90 (m, NH_2-C(=NH)-NH-$(CH_2)_2$-C<u>H</u>$_2$-CH-), 1.73 (br, -O-CH_2-C<u>H</u>$_2$-C<u>H</u>$_2$-CH_2-O), 1.69 (m, NH_2-C(=NH)-NH-CH_2-C<u>H</u>$_2$-CH_2-), 1.56 (br, -C<u>H</u>$_2$-CH2-C<u>H</u>$_2$-CH_2-OH), 1.39 (br, -N-$(CH_2)_2$-C<u>H</u>$_2$-$(CH_2)_2$-OH), 0.88 (t, CH_2-CH_2-C<u>H</u>$_3$).

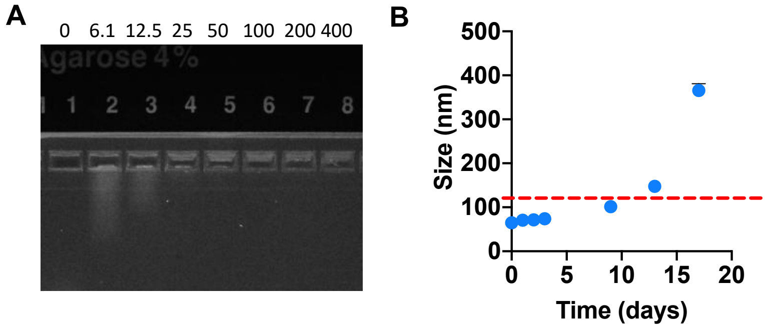

Figure S6: A, Agarose retardation assay of arginine-modified pBAE. Nanoparticles were formed using CpG and arginine-modified pBAE at different w/w ratios and loaded onto an agarose gel to assess CpG mobility by electrophoresis. **B**, Stability study of CpG-NPs in PBS (determined by DLS).

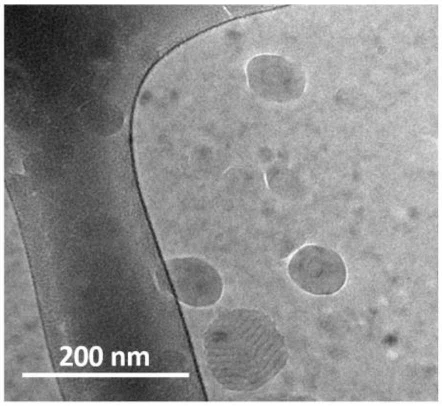

Figure S7: Cryo-Transmission electron microscopy (TEM) images of the CpG-NPs. Scale bar = 200 nm.

Table S1: Biophysical characterization of CpG- and CpC-containing nanoparticles as determined by dynamic light scattering (DLS).

Particle	Size (nm)	Surface charge (mV)	Polydispersity Index (PDI)
CpG-NPs	62.51 ± 0.59	23.1 ± 2.04	0.115 ± 0.02
CpC_{Ctrl}-NPs	59.72 ± 3.8	21 ± 2.24	0.207 ± 0.02

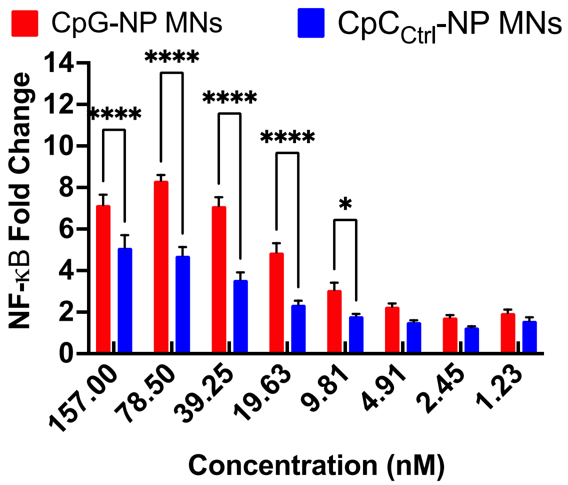

Figure S8: Dose-response of the NF-kB response produced by CpG-NP and CpC-Crt-NP released from the MNs in mouse TLR9 Reporter HEK293 cell line (n = 4 biologically independent samples).

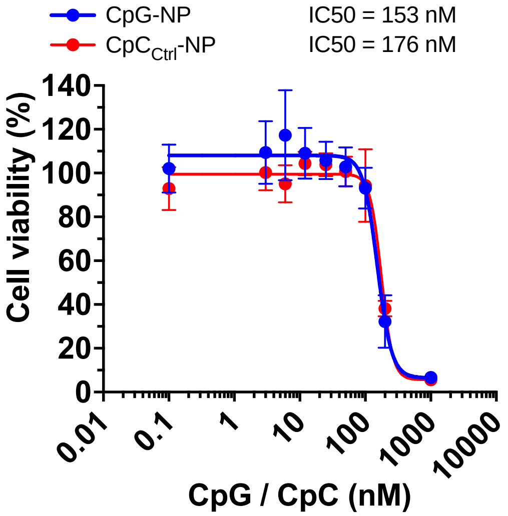

Figure S9: Cell viability profile of different concentrations of CpG-NPs and CpC-Crt-NP were analyzed 24 h post treatment. Samples were normalized to untreated cells. Data are represented as mean ± SD (n = 3).

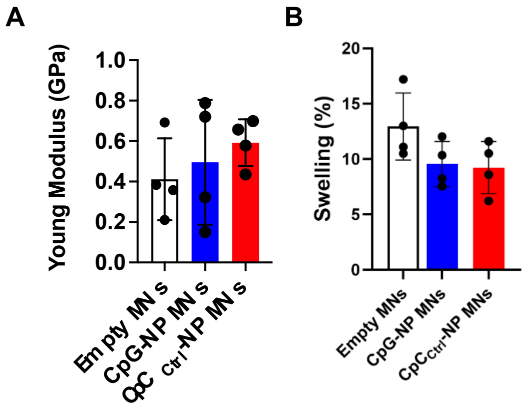

Figure S10: Characterization of the mechanical properties of HA-based MNs. **A**, A compression test was performed to compare the mechanical strength of empty MNs versus CpG-NP-loaded MNs and CpC_{Ctrl}-NP-loaded MNs. Data are means ± s.e.m. (n = 4). **B,** Analysis of the swelling ability *in vitro* by weight measurement. Data are means ± s.e.m. (n = 4).

Table S2: Assessment of CpG-NP release profile *in vivo* when delivered with HA-based MNs by tracking the fluorescence intensity of labeled NPs over time. Data are means ± s.e.m. (n = 4).

MN administration time (H)	% CpG-NPs released
3	-
6	57±18%
24	52±12%

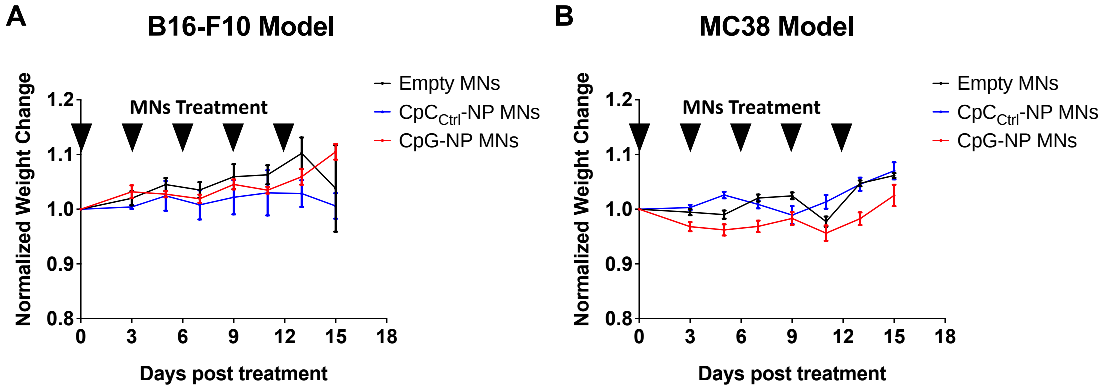

Figure S11: Mice body weight following Empty MNs, CpC_{Ctrl}-NP MNs, and CpG-NP MNs therapy in melanoma B16-F10 model (**A**) and colon MC38 model (**B**). Mice with 20-40 mm^3 tumors were treated five times, 3 days apart. Body weight was measured every other day.

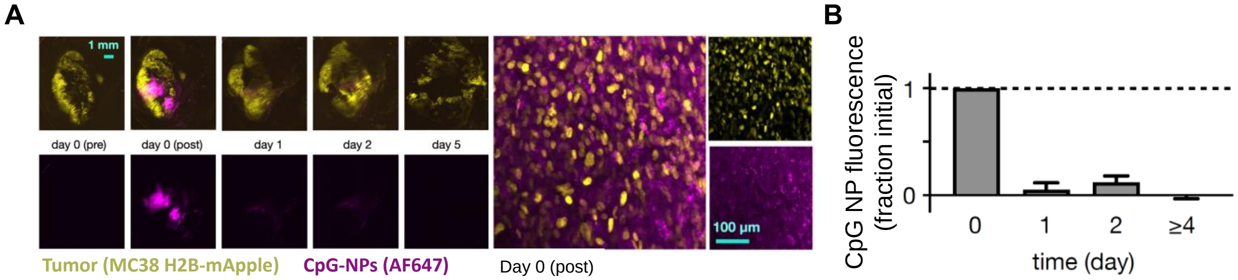

Figure S12: A, IVM of MC38-mApple tumors (yellow) injected fluorescent CpG-NPs intratumorally (magenta) (left, scale bar: 1 mm; right, scale bar: 100 µm). **B**, Quantification of the fluorescence intensity of CpG-NPs within tumor tissue, following intratumoral injection. Data are normalized to fluorescence levels observed immediately after injection (day 0). Data are means ± std. dev. (n = 3).

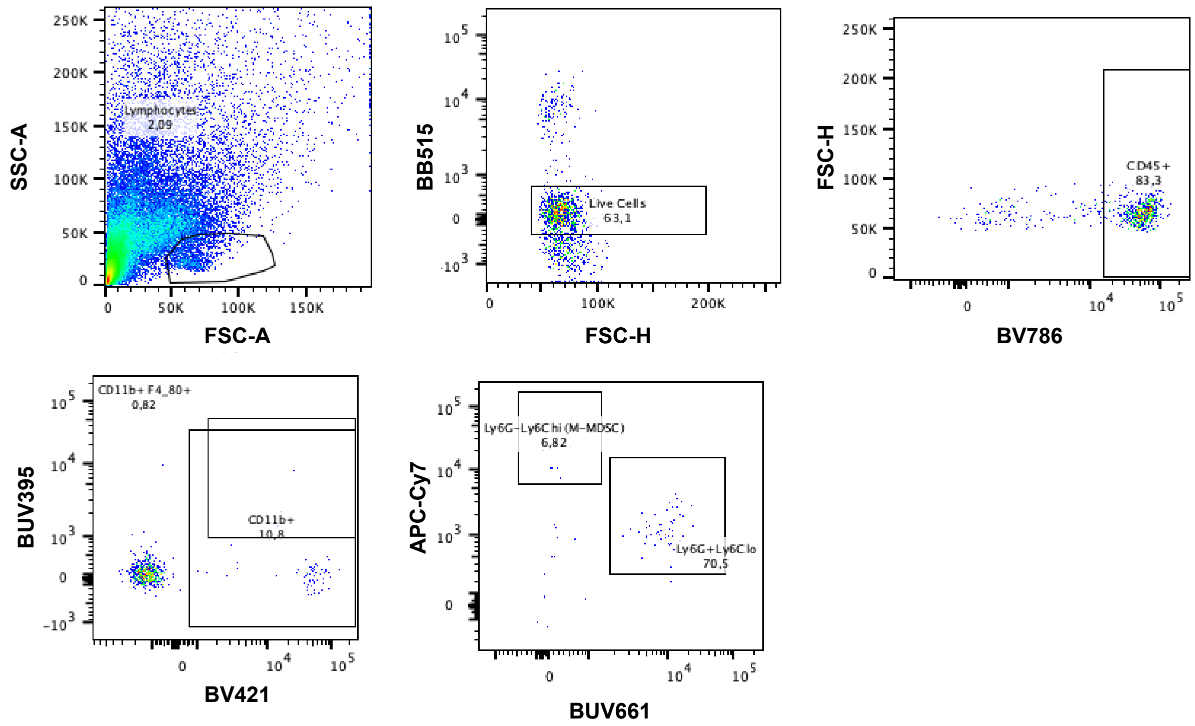

Figure S13: Macrophages gating strategy by flow cytometry when analyzing cells recovered from ISF using microneedles.

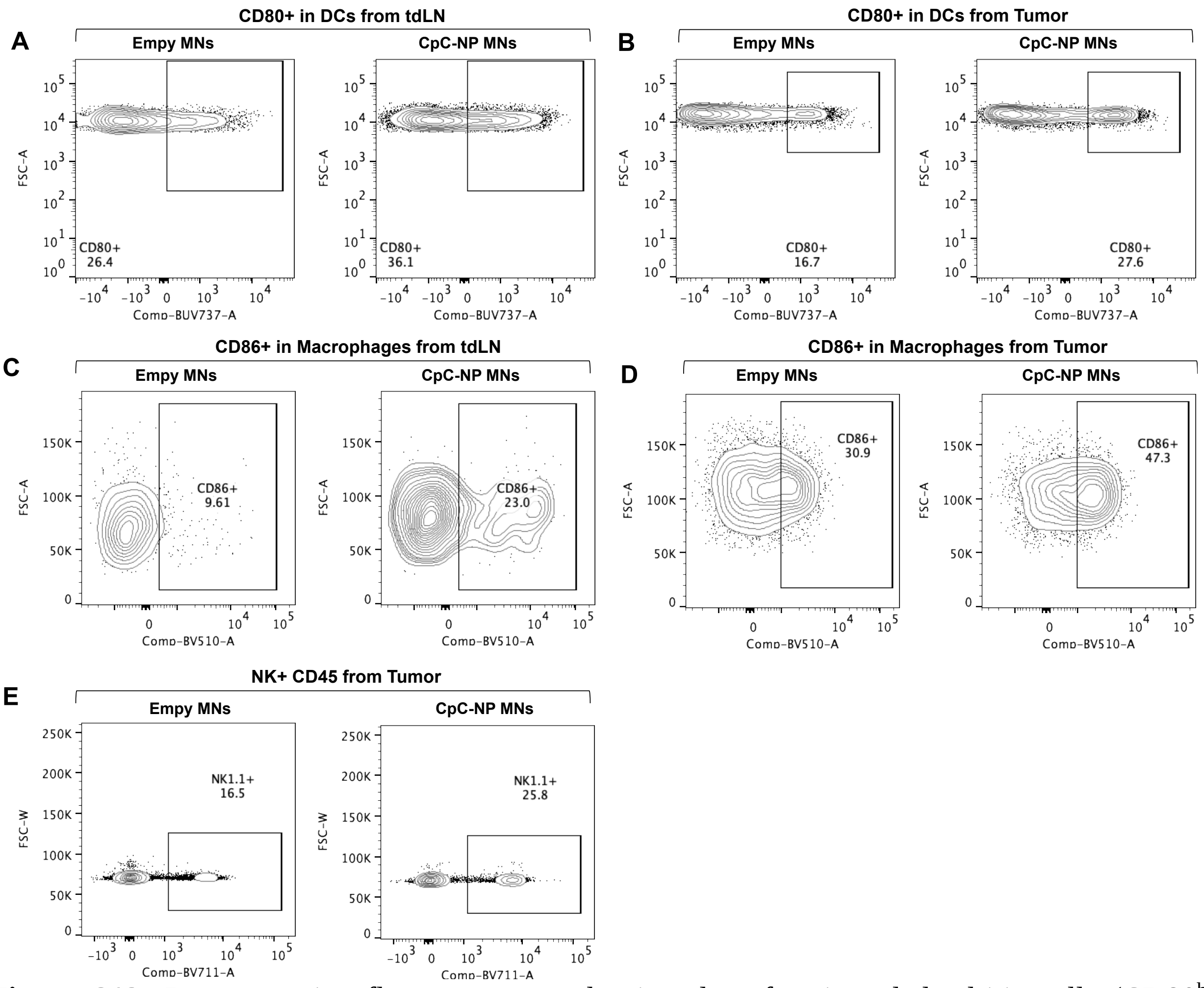

Figure S13: Representative flow cytometry density plot of activated dendritic cells (CD80^{hi} CD11c^{+}MHCII^{+}CD45^{+}) in tdLNs (**A**) and tumors (**B**) 48 h post transdermal delivery of CpG-NPs. **c,d,** Representative flow cytometry density plot of activated macrophages (CD86^{hi} F4/80^{+}CD11b^{+}CD45^{+}) in tdLNs (**C**) and tumors (**D**) 48 h post transdermal delivery of CpG-NPs. **E**, Representative flow cytometry density plot of natural killer cells in tumor lysates 48 h post-treatment with CpG-NPs.

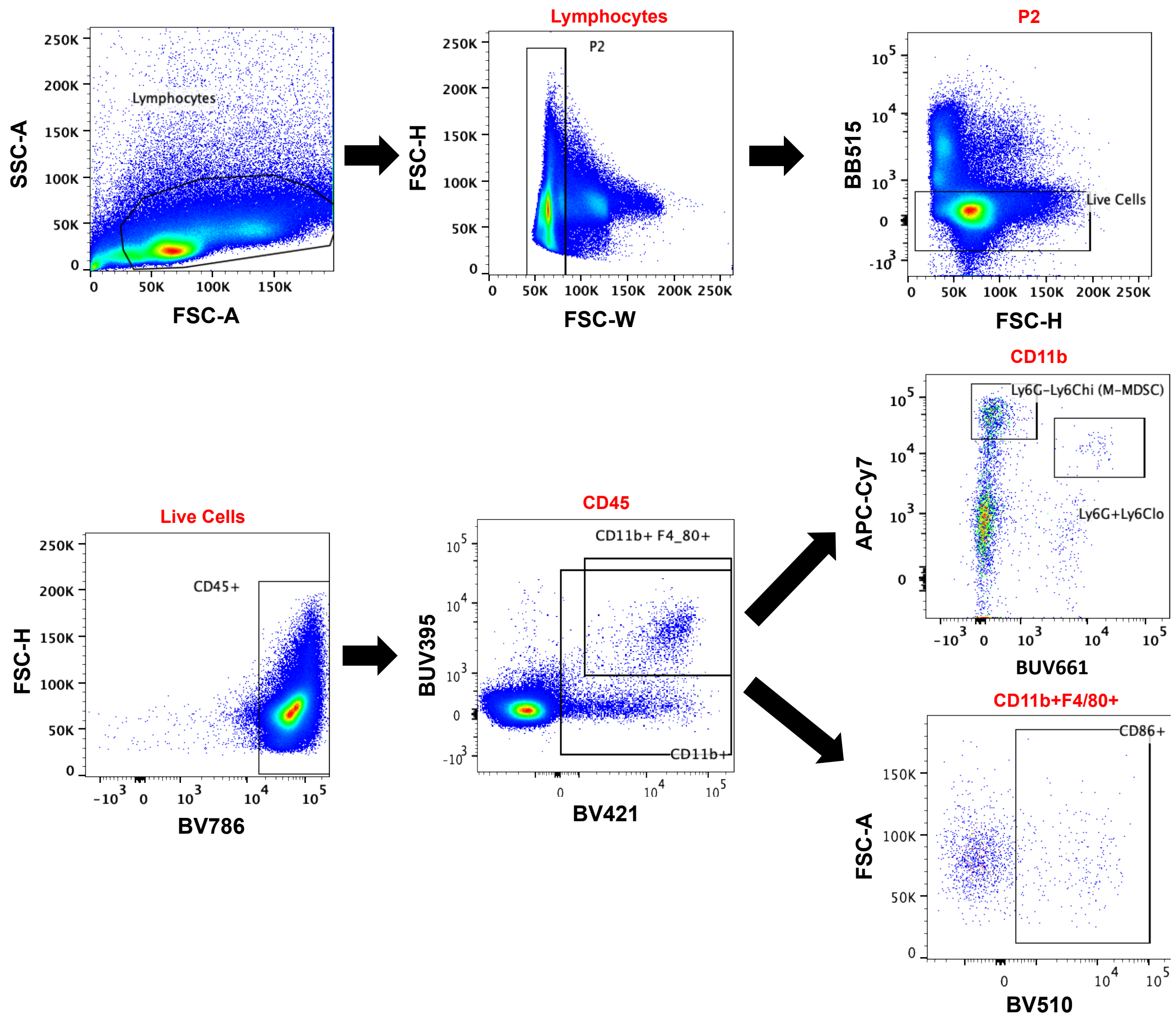

Figure S14: Macrophages gating strategy by flow cytometry.

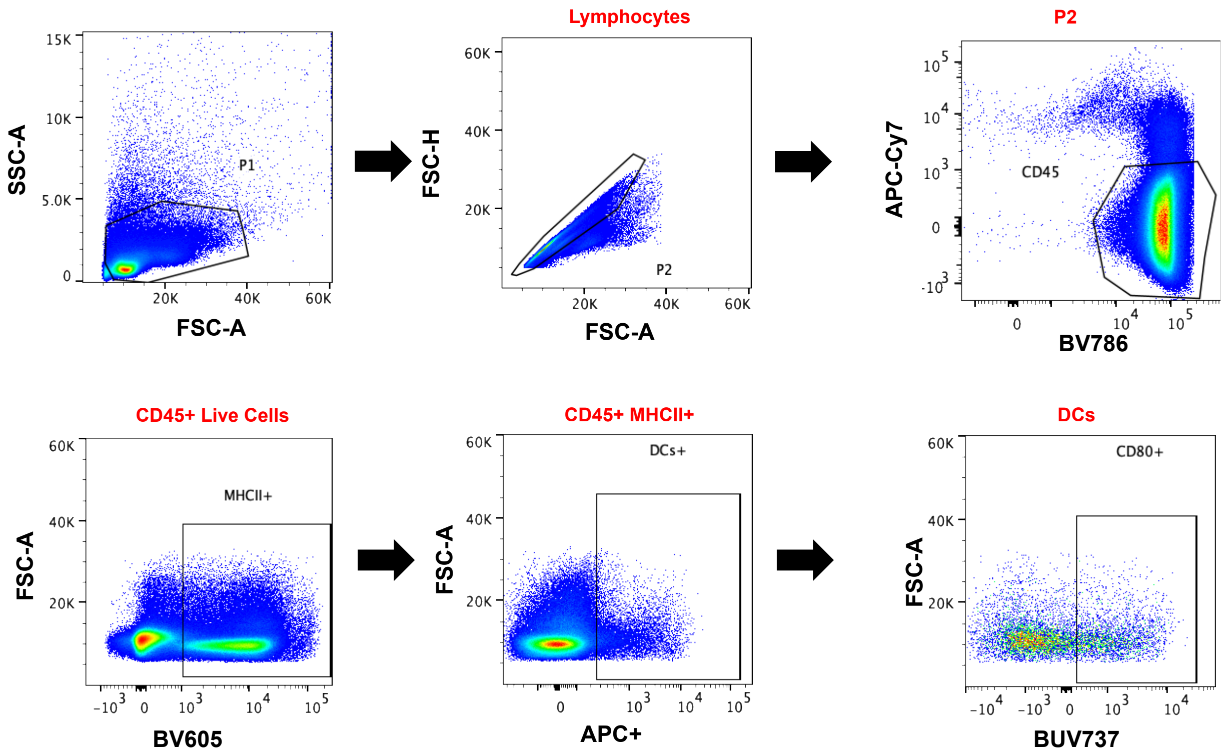

Figure S15: Dendritic Cells gating strategy by flow cytometry.

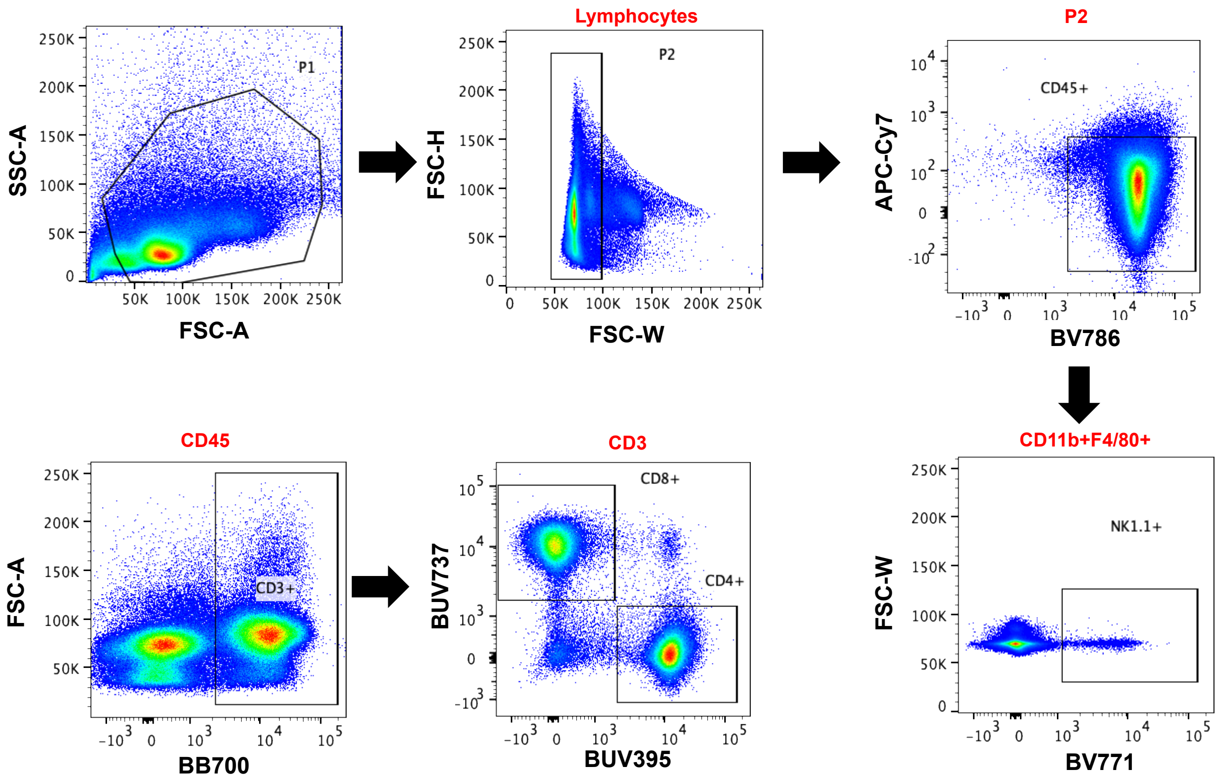

Figure S16: T Cells gating strategy by flow cytometry.
